# Genetic Diversity and Phylogenetic Relationships Among Accessions of *Pediomelum tenuiflorum* (Pursh) A.N. Egan

**DOI:** 10.3390/genes17040490

**Published:** 2026-04-20

**Authors:** Cynthia O. Anukege, Mark Schoenbeck, P. Roxanne Kellar

**Affiliations:** Department of Biology, University of Nebraska at Omaha, Omaha, NE 68182, USA; cynthia.anukege@uga.edu (C.O.A.); mschoenbeck@unomaha.edu (M.S.)

**Keywords:** chloroplast, chloroplast capture, hybridization, introgression, ITS, ISSR, nuclear, reticulate evolution, systematics

## Abstract

Background: Differentiating plant species is complex, complicated by morphological similarities that confound species’ delineation. For hundreds of years, researchers have used herbarium specimens to study plant morphology, and over the last forty years, these samples have also served as material for molecular phylogenetic research. Taxonomists have alternately split and combined morphotypes of *Pediomelum tenuiflorum* for two centuries. With samples of *P. tenuiflorum* from across its distribution, this research aimed to (1) infer a robust phylogeny using molecular data, i.e., gene sequences from chloroplast and nuclear genomes; (2) assess genetic diversity using molecular markers, specifically Inter Simple Sequence Repeats (ISSRs); (3) provide evidence to support the taxonomic placement and possible splitting of *P. tenuiflorum*; and (4) identify consistent morphological characteristics using a correlation matrix to distinguish among the morphotypes. Results: Striking morphological differences among the individuals of *P. tenuiflorum* from across the species’ distribution resulted in more than two morphotypes. Phylogenetic data suggest hybridization is occurring among genetically and morphologically distinct members of *P. tenuiflorum* and with other species in the genus *Pediomelum,* whereas ISSR results indicate detectable genetic variation but do not resolve discrete clusters. This study reports the first ISSR markers used to assess genetic diversity in *Pediomelum* species. Conclusions: Morphological and genetic variation exist across individuals of *P. tenuiflorum* but not in monophyletic groups that support splitting the morphotypes into multiple species. Future investigations into chromosome numbers might reveal polyploidization in the lineage, and phylogenies estimated from low-copy nuclear genes could elucidate hybridization pathways.

## 1. Introduction

Accurate identification of plant species is essential for their use in pharmaceuticals, food, clothing, art, and other applications. For centuries, morphological characters have served as the primary means of distinguishing species, describing diversity, estimating phylogenetic relationships, and classifying organisms into named groups. Researchers identify and classify new plant species by examining their morphological and molecular traits and comparing them to established species [1]. Individuals of the same plant species may exhibit variation that complicates species distinction since the characteristics of flowers, leaves, and roots can vary depending on differing genotypes or divergent phenotypes resulting from abiotic factors including temperature, day length and daylight, and soil conditions [2]. Systematists use new plant collections along with herbarium specimens and common gardens to investigate plant species’ distinctions and characterize intraspecific/interspecific variation.

Approximately 390 million plant specimens are housed in over 3000 active herbaria around the world [3]. These botanical vouchers (containing roots, stems, leaves, flowers, and/or fruits) include multiple representatives of thousands of plant species and are an important resource for comparing morphological characteristics within and among species [4]. Multiple herbarium specimens of a species can be viewed and studied at one time, such that species with consistent morphological differences can be split into separate species or subspecies, and individuals that share morphological variation can be grouped. Such groupings using molecular systematics and variations in flower and leaf morphology were used to revise 29 species of *Psiguria* (Cucurbitaceae) to six species after studying 758 herbarium specimens from six herbaria [5,6]. Leaf tissue samples from herbarium specimens are used for DNA barcoding, phylogenetic studies, and species identification [5,7]; this source is particularly important when the species grows in isolated or hard-to-reach locations or members are difficult to identify [8].

It is not always known if the morphological differences among individuals of a species are based on genetics or environmental pressures, but systematists can use common gardens to investigate the possible source of this variation. Common gardens play an important role in plant identification and classification and have a long tradition in plant evolution studies [9]. In common gardens, morphologically varying individuals of a species are grown in a single location under like environmental conditions that are free from the climatic, biotic, or nutritional stressors of their natural habitats [10]. Growing morphotypes (individuals of a species with different morphologies) from different populations under identical environmental conditions can help botanists determine whether observed morphological differences are based on environmental factors (phenotypic variation), genetic features (genotypic variation), or a combination of both [9,11]. If morphological variation is observed among individuals in a common garden, it may be a result of its genotype alone [12], genotype and environmental interactions [13], or different genotypes producing the same phenotype [10]. These results, in turn, can be used to split species apart or keep them together.

Traditionally, botanists have identified and described plant species based on morphological characteristics of leaves, stems, roots, flowers, and fruits [14]. However, species delineations based on these characteristics can be difficult because flowers and fruits may only be present for a limited time, at different times of the year, and/or in regions with seasons, even leaves may not be present for much of the year [15]. Morphological studies remain essential, especially for field identifications, but morphology alone may be insufficient in taxonomically complex groups, particularly when hybridization is involved [16]. Today’s plant systematist integrates both morphological and molecular data, leveraging both lines of evidence in phylogenetic and taxonomic investigations [17].

The use of molecular sequence data for plant identification and estimating phylogenetic relationships has gained momentum as procedures and technologies have become easier and more cost-effective. DNA, RNA, and protein sequences provide insights into genetic variation and evolutionary processes that are not provided by morphological data [18]. Chloroplast genes (e.g., *rbcL* and *matK*) and intergenic regions [19,20], nuclear ribosomal DNA (nrDNA) and the internal transcribed spacer (ITS) regions, and mitochondrial genes are the most used regions for phylogenetic studies at different taxonomic levels due to their conservation and variability [21]. NrDNA and plastid DNA (cpDNA) have traditionally been used by plant systematists for species distinction and phylogeny reconstruction at or below the genus level [22,23,24,25]. Chloroplast genes, *rbcL* and *matK*, were chosen as the two coding regions to serve as a core barcode for plants to be supplemented by other regions [26], and the variation in noncoding regions of cpDNA serves as an additional source of data for systematics and population genetics [20]. At lower taxonomic levels, these plastid and nuclear markers can provide complementary perspectives on relationships, but they may also reveal conflicting histories when hybridization, introgression, or lineage sorting has occurred. Using noncoding regions of cpDNA to estimate phylogenetic relationships among plant samples began in the early 1990s [27], and ref. [28] explored the use of noncoding cpDNA regions in phylogenetic studies at lower taxonomic levels under the assumption that the noncoding regions are less functional than coding regions and, therefore, have higher levels of variation necessary for distinguishing closely related species.

Serving as part of the transcriptional unit of nrDNA, a ribosomal repeat occurs in all living organisms except vertebrates [29], and sequences of its coding and noncoding regions are used for high and low taxonomic level plant phylogenetic inference, respectively [30]. Two noncoding internal transcribed spacers (ITS1 and ITS2) occur between the 18S and 26S ribosomal regions of the nuclear ribosomal repeat and on either side of the highly conserved 5.8S ribosomal region [31]. The ITS regions have been sequenced for many plant species, and they exist in large numbers in the plant nuclear genome, eliminating the need for cloning [23,24,29,30,32]. Next-generation sequencing (NGS) is a high-throughput sequencing technology that can sequence and analyze entire genomes or targeted regions in a single day [33], at a fraction of the cost, and with increased accuracy and coverage over traditional Sanger sequencing [34]. With the advent of NGS, molecular phylogenies can now be estimated from both whole chloroplast genomes and nrDNA [35].

Genetic diversity metrics quantify the genetic variation among or within populations [36]. To assess genetic variability, molecular methods can be used at the nucleotide level to identify single-nucleotide polymorphisms (SNPs) or at the level of genes to characterize allele frequencies within and among individuals of a species [37]. Inter-Simple Sequence Repeats (ISSRs) can be analyzed for polymorphisms using PCR-based ISSR analysis, where molecular markers are generated from single primer PCR amplifications in which the primers are based on dinucleotide or trinucleotide repeat motifs [38]. ISSRs are found between short, repeated sequences of DNA called microsatellites (simple sequence repeats), and they offer potential in determining inter- and intra-genomic diversity because they reveal variation in regions of the genome that are unique [37,39]. Visible bands observed on electrophoresis gels are defined as dominant markers and are assigned either 1 = present or 0 = absent [38]. ISSRs have become a popular method for accessing hidden genetic diversity in organisms without prior knowledge of their genomes [40], and ref. [41] suggested that ISSRs may be used in delimiting species where other markers have failed to provide resolution.

Together, herbarium data, common-garden observations, phylogenetic markers, and genetic diversity analyses provide a useful framework for evaluating species limits in morphologically variable plant groups. This integrative approach is especially valuable in taxa whose taxonomic boundaries have been unstable over time.

**Figure 1:**
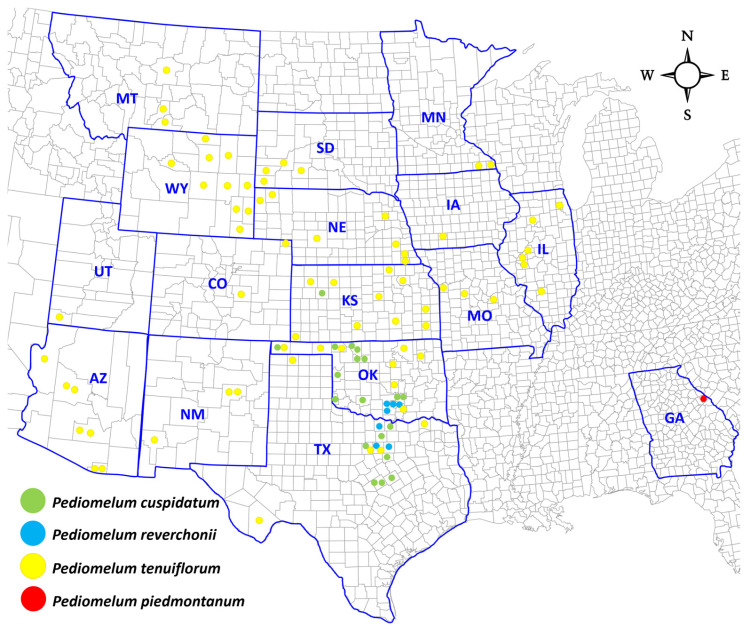
Map of the central United States showing the distribution of *P. tenuiflorum* (yellow dots) and closely related species *P. cuspidatum* (green dots), *P. reverchonii* (blue dots), and *P. piedmontanum* (red dot) and all locations where herbarium specimens were collected. Each dot represents a herbarium specimen borrowed for this study, and each state represents where the herbarium is located. The U.S. base map was retrieved from https://freevectormaps.com/united-states/US-EPS-01-1003 (accessed on 15 March 2024).

*P. tenuiflorum* (Pursh) A.N. Egan, commonly known as slender-flowered scurf-pea, is a flowering, perennial plant species in the legume family, Fabaceae, and is richly distributed across the central United States (Figure 1).

**Figure 2:**
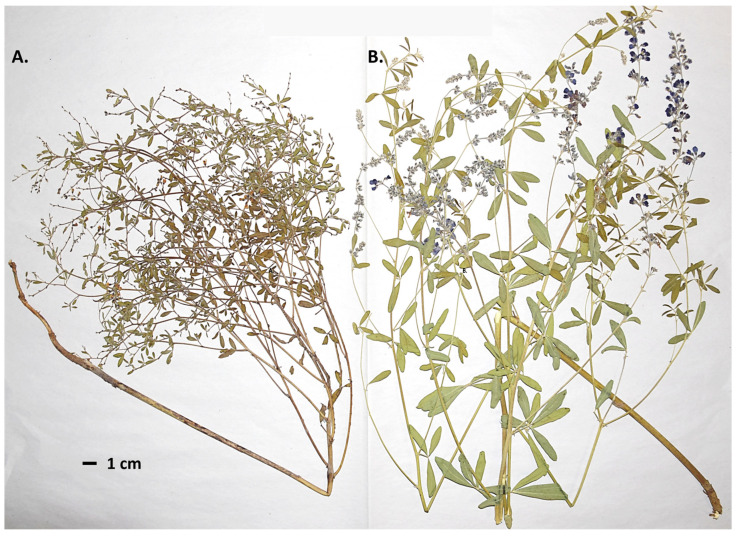
Two morphotypes of *P. tenuiflorum* were observed in Nebraska. The individuals in (**A**), with smaller stems and leaves and fewer flowers, are from central and western Nebraska, and those in (**B**), with larger stems and leaves and more flowers, are from southeastern Nebraska. Photo by P.R.K.

Over the last 200 years, taxonomists have moved *P. tenuiflorum* based on morphological characters to and from different genera—they originally placed *P. tenuiflorum* in the genus *Psoralidium* and have grouped and split *P. tenuiflorum* with *Pediomelum floribundum* [42,43]. At present, these former species are grouped and named *P. tenuiflorum*. *P. tenuiflorum* was moved from the genus *Psoralidium* based on morphological characters and gaps in the DNA sequence data [44], and phylogenetic analyses utilizing DNA markers that revealed its closer relationship to other *Pediomelum* species [45]. Recently, two different morphotypes of *P. tenuiflorum* were identified from disjunct populations in Nebraska (Figure 2; [46]).

Some individuals of the species are smaller with smaller stems, leaves, and flowers, and greater internode lengths, and others are larger with more sturdy stems, pronounced leaves, more flowers, and shorter internode lengths [46]. Ref. [46] suggested that the two morphotypes may be two different species based on statistically significant morphological differences among groups of individuals in multiple floral and vegetative measurements, but they only studied samples from Nebraska. We found no studies published about the breeding system of *P. tenuiflorum*; floristic information indicates that the species is presumed to be insect-pollinated, with no specific pollinator or selfing/outcrossing data reported.

Ref. [46] estimated a phylogeny including these morphotypes based on five chloroplast regions and the nuclear ITS1 and ITS2 regions, which showed resolution but did not have bootstrap support to distinguish their three samples of *P. tenuiflorum*. To test whether the morphological differences in the species were a result of environmental conditions or had a genetic component, ref. [46] planted a common garden using seeds of *P. tenuiflorum* from eight locations across Nebraska and Julesburg, Colorado. In 2021, 72 seeds were planted, and 37 individuals survived through 2024. An investigation of the morphological variation among the common garden individuals could help determine if the two morphotypes should be split into different species or not. If individuals in the common garden from the varying environments had morphological characteristics that were the same, this result would support the hypothesis that the morphological differences seen in the field are due to phenotypic variation; however, if the common garden individuals had the same morphological characteristics as they do in their natural environments, this result would support the hypothesis that the morphological variation is completely or at least partially genetically based.

*P. tenuiflorum* was chosen for this investigation because it presents a long-standing taxonomic problem: morphologically divergent populations have been treated inconsistently, and it remains unclear whether this variation reflects phenotypic plasticity, genetic differentiation, or both. Clarifying species limits in such a variable taxon is important for both systematics and conservation, because accurate delimitation influences how biodiversity is recognized and how populations of concern are evaluated and managed.

In this project, we investigated the morphological and molecular variation among individuals of *P. tenuiflorum* to determine if the variation occurs in evolutionarily distinct lineages. To do so, we integrated evidence from herbarium specimens, a common garden, chloroplast and nuclear phylogenetic data, and ISSR markers sampled across the species’ broader geographic distribution.

## 2. Materials and Methods

### 2.1. Sample Collection

One hundred and one (101) herbarium specimens were borrowed from 13 herbaria across the U.S., including the following number (in parentheses) of collections: *P. tenuiflorum* (71); *P. reverchonii* (10), *P. cuspidatum* (20), and *P. piedmontanum* (1). With permission, leaf tissue was harvested from 41 of the herbarium specimens and obtained from 30 of the common garden individuals for DNA studies (Table S1).

### 2.2. Morphological Measurements

The largest stem, including all branches, leaflets, flowers, and fruits, was collected from every individual in the common garden by P.R.K. in June of 2023, before being pressed and dried. From 30 of these collections, measurements were recorded in millimeters (mm), including corolla length, stem diameter and length, leaflet width and length, and flower internode length. Stem diameter was measured using a digital micrometer (Dicfeos), and it was measured 15 cm from the base of the stem. All other characters were measured with a metric ruler under a dissecting microscope. Stem length was measured from the base to the tip of the longest primary stem. Corolla length was measured only on fully developed flowers from the base of the corolla to the tips of the wing petals. Rachis length was the measurement of internodes only on fully developed inflorescences. Leaflet lengths and widths were recorded for every leaflet on every collection.

### 2.3. Statistical and Data Analysis

The morphological data were analyzed using R (4.4.0) in RStudio (2024.12.1+563). Before analysis, rows that had missing values across the measured traits were removed using (na.omit), a listwise deletion. To assess correlations among the morphological characters between the morphotypes, Pearson correlation coefficients were calculated in a pairwise fashion for all characters using the cor () function and visualized on a matrix (heat map) using heatmap.2 () and ggplot2 package. Each cell of the matrix contains a correlation coefficient. Positive correlations indicate that as one measurement increases, the other increases, and negative correlations indicate that as one measurement decreases, the other decreases. Darker tones of red or blue indicate stronger correlations.

### 2.4. DNA Extraction

Total genomic DNA was extracted from 147 samples of *Pediomelum* across the U.S. distribution using 0.02 g of dried leaf tissue per sample and the IBI Plant Genomic DNA Mini Kit (IBI Scientific, Dubuque, IA, USA) following the manufacturer’s protocol, except during lysis, samples were incubated for one hour, inverting the tubes every 15 min, and DNA was eluted twice—first with 50 µL and then with 30 µL of elution buffer. Each extraction was quantified using a Qubit 2.0 or 4.0 fluorometer (ThermoFisher Scientific, Waltham, MA, USA).

### 2.5. Library Preparation and Sequencing

Seventy-one of the DNA extractions discussed above were selected for library preparation based on funds available and the desire to include samples from as many different geographic locations as possible. DNA libraries were prepared using 30 µL of each DNA extraction, using the Illumina DNA Prep Kit (Illumina, Inc., San Diego, CA, USA), following the Illumina DNA Prep reference guide. Library preps were quantified on the Qubit 4.0 fluorometer for DNA quantities of 1–500 ng or higher. Library preps were qualified for fragment sizes ranging from 360 to 700 bp using an Agilent TapeStation 4150 (Santa Clara, CA, USA). Library preps were pooled and then sequenced using paired-end sequencing on an Illumina NextSeq 550 System (RRID:SCR_016381) at the University of Nebraska at Omaha.

### 2.6. Sequence Assembly and Phylogenetic Tree Estimation

FASTQ files were downloaded from the Illumina sequencer and uploaded into Geneious 10.2.6 software (https://www.geneious.com). Consensus sequences of whole chloroplast genomes and nuclear ITS1 and ITS2 were compiled for each sample using reference-based mapping. Reads were mapped to reference sequences of *P. tenuiflorum* (cpDNA accession number MN115428 and ITS1 + 5.8S + ITS2 accession number MN058282); ref. [46] downloaded from GenBank [47]. Consensus sequences were aligned in Geneious using the sequence alignment tool MAFFT (v. 7.450C); with default algorithm, scoring matrix: 200 PAM/k = 2, gap open penalty: 1.53, and offset value 0.123; [48,49]. All genes were annotated, and small sequence alignment errors were deleted or changed to Ns. Maximum likelihood (ML) analyses were conducted using RAxML, version 8 [50], accessed through Geneious. For rooting the phylogenetic analyses, *Pediomelum argophyllum* was selected as the outgroup. Previous phylogenetic studies of *Pediomelum* identified *P. argophyllum*, *P. cuspidatum*, *P. reverchonii*, *P. esculentum*, *P. digitatum*, and *P. piedmontanum* as species outside the focal *P. tenuiflorum* lineage that could serve as potential outgroups [44,46]. We selected *P. argophyllum* because it was the most distantly related species among the candidate outgroup taxa available to us. ML analyses used rapid hill-climbing starting with a random tree. Likelihood scores of the optimal trees were generated by RAxML using the CIPRES Science Gateway (www.phylo.org). Nonparametric bootstrap (BS) analyses on 1000 replicates were also performed by RAxML using CIPRES, and BS consensus trees were assembled in Geneious. Phylogenetic tree figures were edited for publication using Adobe Illustratorv 29.2 [51]. To test for incongruence between the cp genome and ITS phylogenetic hypotheses, an approximately unbiased (AU) topology test was conducted in IQ-TREE. The maximum-likelihood ITS tree was evaluated against the plastid genome alignment using 10,000 RELL replicates.

### 2.7. Inter-Simple Sequence Repeats Analysis

ISSR amplification products were used as a rapid and relatively low-cost exploratory approach for detecting polymorphism in *Pediomelum*, particularly because this method does not require a priori knowledge of the genome’s SSR composition or structure. Forty-nine ISSR primers were tested and validated for their suitability in DNA amplification of *P. tenuiflorum*. These primers were originally selected for their ability to amplify markers in *Amorphophallus* species. Each PCR cocktail was prepared using a buffer containing Taq polymerase, molecular-grade water, 1.5 mM magnesium chloride (MgCl_2_), 20 mM Tris hydrogen chloride (HCl_2_) with a pH of 8.4, 50 mM potassium chloride (KCl_2_), and 0.08 mM each deoxynucleotide triphosphate. Eleven primers (three dinucleotide and eight trinucleotide) were selected as follows: AC_8_G, AG_8_, AG_8_C, AGT_6_G, CTC_6_A, CTC_6_C, GTT_6_T, TC_8_A, TGC_6_, TGC_6_C, and TGC_6_G.

Twenty samples of *P. tenuiflorum* were selected for ISSR analysis, including 10 samples from the common garden and 10 from locations across the species’ geographic distribution (S). Each sample was normalized to a final concentration of 70 ng/µL for PCR. PCR amplifications were conducted in 50 μL reactions using a Techne Thermal Cycler (GMI, Ramsey, MN, USA). Reaction conditions were as follows: initial denaturing at 94 °C for 6 min, followed by 32 cycles of 94 °C for 1 min, 50 °C for 1 min, and 72 °C for 2 min, with final elongation at 72 °C for 7 min. Amplicons were stained with SYBR green loading dye (Bio-Rad, Hercules, CA, USA). All gels were 1.5% agarose SB (sodium borate; [52]), and samples were electrophoresed at 150 to 154 volts. The molecular ladder used for screening was a 1000 base pair (bp) ladder, which was then changed to a TrackItTM 100 bp DNA ladder (Invitrogen, ThermoFisher Scientific, Waltham, MA, USA) for all amplifications because the 1000 bp ladder did not show bands.

## 3. Results

### 3.1. Morphological Characters

**Figure 3:**
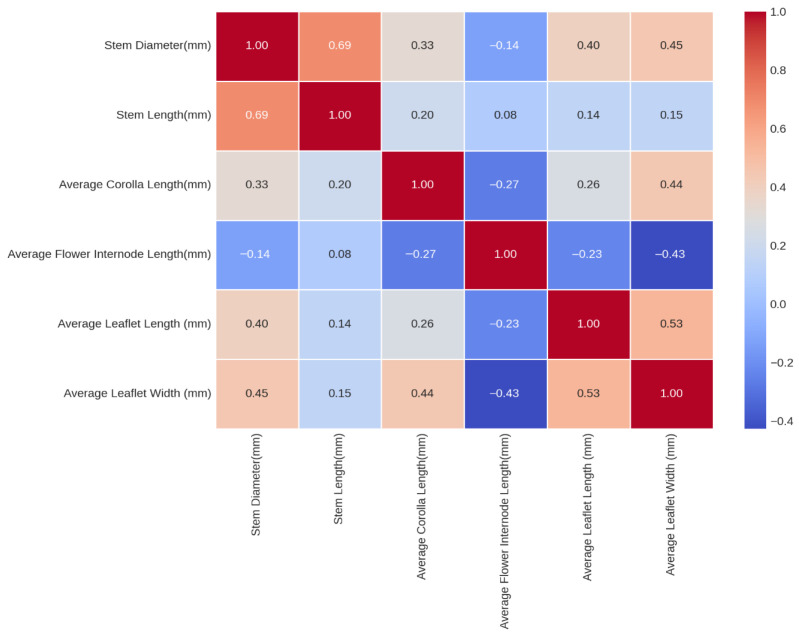
Correlation matrix of six morphological characters measured on 30 samples of *P. tenuiflorum* from the common garden. Matrix generated using a heatmap to highlight the correlation strengths (darker tones = stronger; lighter tones = weaker). The values in the cells are correlation coefficients (positive values = positive correlations; negative values = negative correlations).

Measurements of six morphological traits (shown in Figure 3) for 30 plant specimens from the common garden were made. Five to 300 measurements per plant were recorded, depending on the traits measured. Nine plant samples lacked corolla length measurements because their flowers had already developed into seeds, and five samples lacked stem diameter measurements when a full branch was not available. The average value for each measurement per sample was calculated (Table S2). Weak negative correlations (Figure 3) were calculated between average flower internode length (AFIL) and stem diameter (−0.14), AFIL and average corolla length (−0.27), and AFIL and average leaflet length (−0.23), and a moderate negative correlation between AFIL and average leaflet width (−0.43). These correlations match the two morphotypes observed in Nebraska. The common garden specimens whose seeds were collected from the intersection of Hwy 737 & Hwy 50 in southeastern NE, Stanton, NE, and Julesburg, CO had the greatest morphological variation among the individuals from the same site. These common-garden data do not by themselves constitute a formal test of discrete morphological clustering, but they do indicate that morphological differences persist under uniform conditions.

### 3.2. Sequence Assembly and Alignment

Complete chloroplast genomes (cpDNA) and ITS1 + 5.8S + ITS2 (ITS) were assembled for 71 *Pediomelum* samples. In regions where quality (Q) scores were less than Q30, bases were replaced with Ns. These replacements totaled less than 0.5% of the overall sequences, and none of the replacements occurred in coding regions, tRNAs, or rRNAs. Mapping sequence reads to the reference genomes resulted in average depths of coverage per base ranging from 218× to 17,822× for cpDNA and from 399× to 39,877× for ITS (Table S3). Sequences of cpDNA and ITS for six additional *Pediomelum* samples [46] were downloaded from GenBank as follows (accession numbers in parentheses): *P. argophyllum* (Jones 27 NE; cpDNA MN115426; ITS MN058286), *P. tenuiflorum* (Sutherland 17 NE; cpDNA MN115428; ITS MN058282), *P. esculentum* (Ahrendsen 14 NE; cpDNA MN115430; ITS MN058283), *P. tenuiflorum* (Sutherland 44 NE; cpDNA MN115431; ITS MN058284), and *P. digitatum* (Jones 22 NE; cpDNA MN115424; ITS MN058285), and *P. tenuiflorum* (no identifier; cpDNA MN115425; ITS MN058281). The following three alignments were made: cpDNA (alignment length [AL] = 128,078 base pairs [bp]; pairwise identity [PI] = 99.1%), cpDNA coding regions (86 genes; AL = 67,140 bp; PI = 99.8%), and ITS (AL = 622 bp; PI = 99.69%). Sequence alignments were uploaded to Dryad (www.datadryad.org; alignments available from the Dryad Digital Repository [doi: 10.5061/dryad.n8pk0p397]). All new DNA sequences were uploaded to GenBank (accession numbers in Table S4).

### 3.3. Phylogenetic Analyses

**Figure 4:**
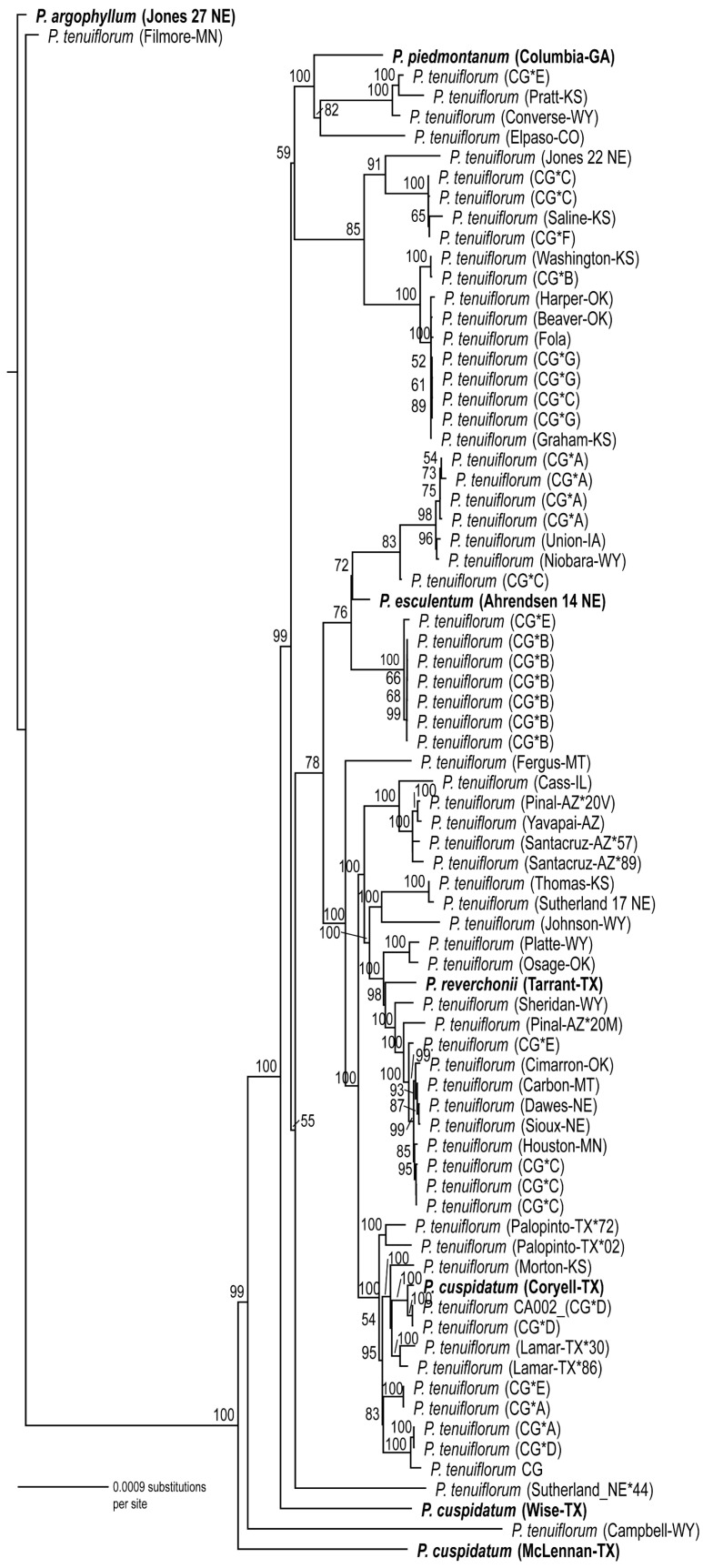
Phylogeny of *P. tenuiflorum* estimated from complete chloroplast genomes. Maximum likelihood (ML) phylogeny (-ln L = 198,603.70) includes 77 samples with *P. argophyllum* as the outgroup. Species other than *P. tenuiflorum* are in bold text. Collection locations (county–state) are shown in parentheses for each sample. Samples from the common garden are indicated with CG* and the Nebraska (except one) location from which their seeds were collected, as follows: A = Nine-mile prairie, B = Stanton County, C = Intersection of Hwy 737&50, D = Table Rock Wildlife Management Area, E = Julesburg, Colorado, F = Jeffrey Reservoir (upper), and G = Jeffrey Reservoir (near). Samples from the same location and county are indicated by * and the last two digits of the collector number, and if they have the same last two digits, the first letter of the collector’s last name is included. Numbers above branches indicate ML bootstrap (BS) values resulting from 1000 replicates. If no BS values are shown, BS ≤ 50.

**Figure 5:**
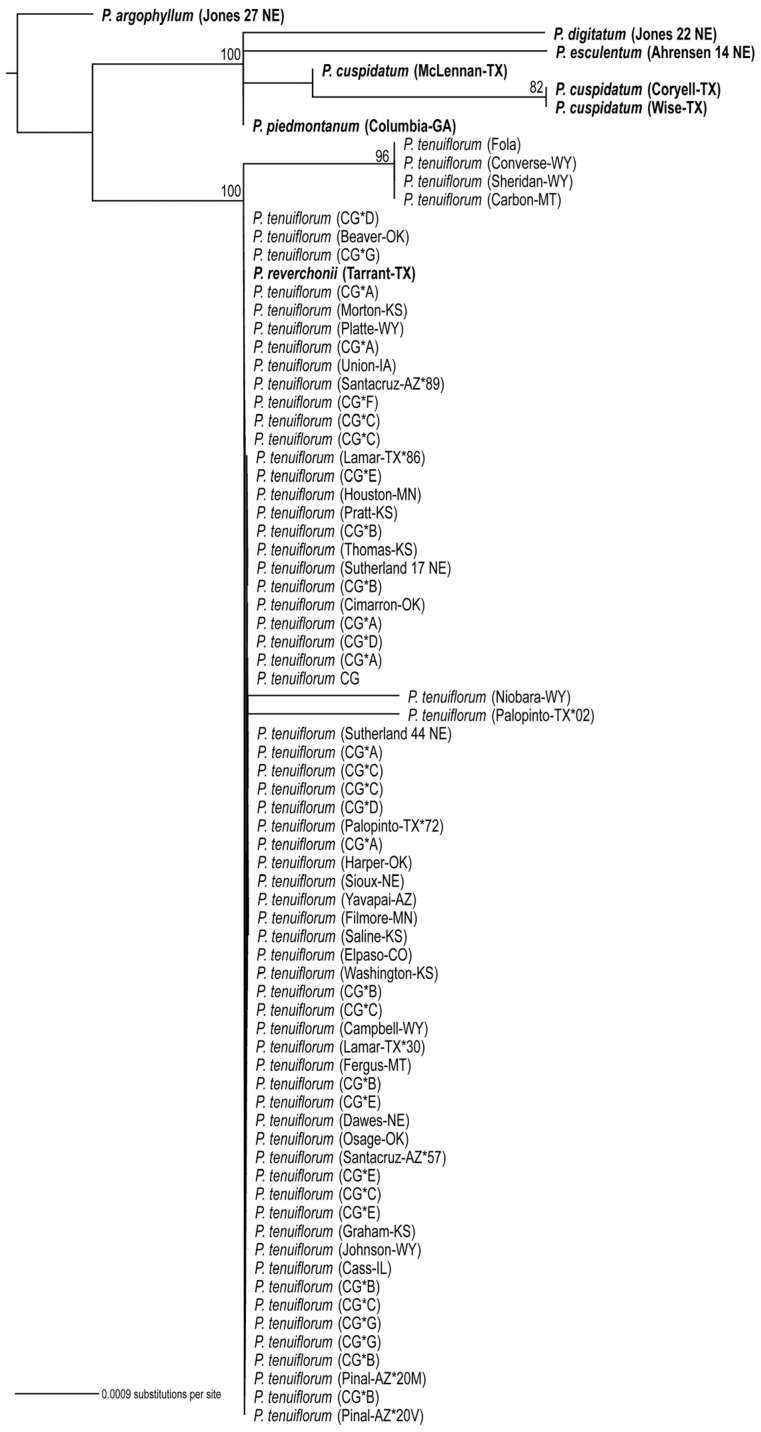
Phylogeny of *P. tenuiflorum* estimated from ITS1 + 5.8S + ITS2. Maximum likelihood (ML) phylogeny (-ln L = 946.49), which includes 77 samples with *P. argophyllum* as the outgroup. Species other than *P. tenuiflorum* are in bold text. Collection locations (county–state) are shown in parentheses for each sample. Samples from the common garden are indicated with CG* and the Nebraska (except one) location from which their seeds were collected, as follows: A = Nine-mile prairie, B = Stanton County, C = Intersection of Hwy 737&50, D = Table Rock Wildlife Management Area, E = Julesburg, Colorado, F = Jeffrey Reservoir (upper), and G = Jeffrey Reservoir (near). Samples from the same location and county are indicated by * and the last two digits of the collector’s number, and if they have the same last two digits, the first letter of the collector’s last name is included. Numbers above branches indicate ML bootstrap (BS) values resulting from 1000 replicates. If no BS values are shown, BS ≤ 50.

Maximum likelihood (ML) trees were estimated for each data set separately: cpDNA (Figure 4), cpDNA coding regions (Figure S1), and ITS (Figure 5). All trees were rooted with *Pediomelum argophyllum*. The topologies of the trees estimated from chloroplast DNA (Figure S1 and Figure 4) are similar, with all but one sample forming a clade with strong bootstrap support (BS = 100). This clade includes all the species of *Pediomelum* in the data set (except for the one sample mentioned above), with species other than *P. tenuiflorum* nested within and among various *P. tenuiflorum* samples and clades, also with strong bootstrap support. In some cases, samples from the same geographic region grouped together; for example, four of the five samples from Arizona form a clade (BS = 100), but in most cases samples from the same geographical regions did not form monophyletic groups, including some samples from the common garden whose seeds were collected from the same population (e.g., samples labeled “CG*A” from Nine-mile Prairie). Some clades collapsed into polytomies, such as six common garden samples whose seeds were collected from Stanton, NE (labeled “CG*B”), but one of these samples also came out in a distant clade and sister to a sample from Washington County, Kansas.

The topology of the ITS tree (Figure 5) is very different from that estimated from cpDNA. In the ITS tree with *P. argophyllum* as the outgroup, all the *P. tenuiflorum* samples form a clade (BS = 100) that is sister to a clade (BS = 100) containing all the other species of *Pediomelum* included in this study, with one exception. *Pediomelum reverchonii* (Tarrant County, TX) is nested within the *P. tenuiflorum* clade. The cpDNA genome alignment significantly rejected the ITS topology in the AU test (*p* = 1.4 × 10^−5^).

### 3.4. Inter-Simple Sequence Repeat Amplifications

**Figure 6:**
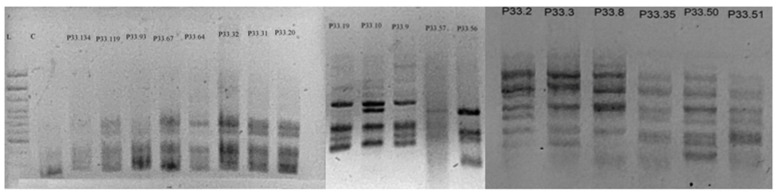
Gel image of ISSR amplification of *P. tenuiflorum* using primer CTC_6_A. Each lane corresponds to a single *P. tenuiflorum* sample, including individuals from the common garden and from across the geographic distribution. Primer CTC_6_A produced strong amplification in most samples and yielded the highest number of scorable bands among all primers tested.

The 11 ISSR primers (Table S5) generated 346 scorable bands ranging from 100 to 2000 bp, of which 61 were polymorphic, yielding an overall polymorphism of 17.68% and indicating a relatively low level of detectable genetic variation among *Pediomelum* individuals. The gel lanes were converted to linear plots in ImageJ 1.54g, and bands were scored by identifying peaks or clusters exceeding 5% of the signal intensity threshold (Figures S2–S9). Primer CTC_6_A produced the greatest total number of amplified bands (Figure 6), whereas primer AC_8_G yielded the highest proportion of polymorphic bands (8 of 21 bands) and was, therefore, used to amplify a subset of twelve samples. In contrast, primer TGC_6_C generated 37 bands but only three polymorphic loci. Across all primers, the number of bands per primer ranged from 0 to 63, and percent polymorphism ranged from 0% to 38.10%, revealing substantial variation in amplification success and informativeness among dinucleotide versus trinucleotide primers and between anchored and unanchored designs, with the most productive primer being an anchored dinucleotide and the least productive (no amplification) being an unanchored trinucleotide. The ISSR component of this study was intended as an exploratory screen for detectable polymorphism rather than a definitive analysis of population structure. Accordingly, although the markers revealed some variation, the limited sample size and marker type constrain the strength of inferences about clustering among lineages.

## 4. Discussion

*P. tenuiflorum* exhibits striking morphological and genetic complexity that cannot be cleanly partitioned into discrete, evolutionarily independent lineages with the markers used in this study. Continuous morphological variation, limited nuclear ITS resolution, and strongly supported chloroplast topologies suggest hybridization, chloroplast capture, and ongoing gene flow across geographic regions and morphotypes [44,45,53,54,55,56].

### 4.1. Morphological Variation and Taxonomic Implications

The common garden experiment showed that the morphological variation previously documented in the field (e.g., internode length, stem robustness, leaflet size; Figure 2) persists under uniform environmental conditions, indicating at least a partial genetic basis to the observed morphotypes [9,10,12,13,46]. Weak to moderate negative correlations between average flower internode length and stem diameter, corolla length, and leaflet dimensions mirror the original Nebraska morphotypes described by [46], but the continuous nature of this variation and the absence of discrete morphological clusters argue against splitting *P. tenuiflorum* based solely on these traits [14,46].

Herbarium collections and the taxonomic history of the group support this interpretation. Nearly two centuries of alternating splitting and lumping of *P. tenuiflorum* and related taxa, including its historical treatment in *Psoralidium* and proposals to recognize *Psoralidium floribundum*, underscore how continuous morphological gradients can obscure species boundaries [42,43,44,45,46]. Although our examination of herbarium specimens across the species’ distribution suggested at least four morphotypes, these did not show consistently non-overlapping character combinations. These results indicate that morphology alone is insufficient for robust species delimitation in this complex [2,16,17,57,58].

### 4.2. Cytonuclear Discordance Between ITS and Chloroplast Phylogenies

The ITS phylogeny (Figure 5) recovered *P. tenuiflorum* as a single, well-supported (BS = 100) clade with little internal resolution, whereas the chloroplast phylogenies (Figure 4 and Figure S1) provided greater resolution and placed multiple *Pediomelum* species within the *P. tenuiflorum* clade. This contrast indicates strong cytonuclear discordance and suggests that our phylogenetic markers captured different aspects of the evolutionary history of the group.

In the ITS tree, the lack of resolution among *P. tenuiflorum* samples suggests shallow divergence, ongoing or recent gene flow, incomplete lineage sorting [56], concerted evolution across nrDNA arrays, or some combination of these [22,23,24,29,30], all of which can homogenize ITS signals across populations and morphotypes. The placement of a morphologically verified *P. reverchonii* specimen within the *P. tenuiflorum* ITS clade is most consistent with nuclear introgression and hybridization rather than misidentification, highlighting that species boundaries among some *Pediomelum* taxa may be weak [44,45,53,54,55].

In contrast, the phylogenies inferred from whole chloroplast genomes or only plastid coding regions provided high resolution among samples and strongly supported a large clade containing nearly all *P. tenuiflorum* individuals, with *P. cuspidatum*, *P. digitatum*, *P. esculentum*, *P. reverchonii*, and *P. piedmontanum* nested within it. The repeated nesting of other *Pediomelum* species inside the *P. tenuiflorum* chloroplast clade, together with the absence of morphotype- or locality-specific monophyletic groups, is best explained by historical and possibly ongoing chloroplast capture accompanied by backcrossing [21,27,53,54,55]. Hypothetically, the hybridization between *P. tenuiflorum* and congeners, followed by repeated backcrossing into predominantly *P. tenuiflorum* nuclear backgrounds, would generate individuals with largely *P. tenuiflorum* nuclear genomes but chloroplast haplotypes derived from other species.

As the focus of our investigation was *P. tenuiflorum*, this species was represented by more accessions than the other congeners included in the phylogenies, and sparse sampling can affect apparent topological placement and the recovery of monophyletic groups. This limitation is most relevant for *P. piedmontanum*, which was represented by a single accession, and its position should therefore be interpreted cautiously. However, the repeated placement of multiple accessions of *P. cuspidatum* and *P. reverchonii* within or among *P. tenuiflorum* lineages suggests that the observed chloroplast discordance is unlikely to be an artifact of sample-size imbalance alone, although broader sampling of congeners will be important for refining the extent and direction of introgression.

The differing results from the ITS and chloroplast data also reflect differences in marker properties. ITS may distinguish deeper lineages but may have limited power to resolve recent reticulate diversification when concerted evolution and gene flow reduce variation [22,23,24,30]. By contrast, whole chloroplast genome data can provide a stronger signal at shallow taxonomic scales [19,20,26,28,35] and can reveal organellar introgression because plastids are typically uniparentally inherited and effectively nonrecombining [21]. Although incomplete lineage sorting may also contribute to the discordance observed here, the strongly supported, species-mixed chloroplast clades and the placement of *P. reverchonii* within the *P. tenuiflorum* ITS clade favor a model in which hybridization and backcrossing are likely the central drivers of the pattern.

### 4.3. ISSR Diversity and Lack of Clustering

ISSR markers detected relatively low polymorphism across individuals (17.68% of bands), yet the presence of variable bands confirms underlying genotypic diversity within *P. tenuiflorum* [37,38,39,40,41]. No obvious clustering was apparent from the ISSR banding patterns, although formal clustering analyses were not performed, and the ISSR dataset was exploratory in scope. It is also possible that some primers were suboptimal for differentiating closely related lineages within *P. tenuiflorum*, underscoring that dominant multi-locus markers, while useful for detecting polymorphism, lack the resolution needed to parse complex reticulate histories [37,38,39,40,41].

### 4.4. Synthesis and Future Directions

Collectively, the morphological, ITS, chloroplast, and ISSR data reveal *P. tenuiflorum* as a morphologically heterogeneous, genetically interconnected complex embedded within a network of historical and ongoing hybridization with closely related *Pediomelum* species. The failure to recover monophyletic groups composed of a single morphotype or locality, and the conflicting signals between nuclear and plastid markers, argue against recognizing narrowly defined, morphotype-based species at this time and instead support treating *P. tenuiflorum* as a single, broadly circumscribed species with extensive intraspecific and interspecific gene flow.

Future work should focus on multi-locus or genome-scale nuclear data (e.g., target capture of low-copy nuclear genes, RADseq, or shallow whole genome resequencing) to disentangle reticulate histories and estimate the direction and timing of hybridization events [25,33,34,35,36]. Cytogenetic studies, including chromosome counts and assessments of ploidy variation, could test whether polyploidy has contributed to the diversification and morphological differentiation within *P. tenuiflorum*, as is common in many legume lineages [18,21,37]. Integrating ecological and geographic data with genomic analyses may also clarify which chloroplast lineages or morphotypes are associated with specific habitats or environmental gradients [1,10,12,36], thereby improving our understanding of how hybridization and gene flow shape diversity in this group.

## 5. Conclusions

This study provides the first broad geographic assessment of morphological and molecular variation in *P. tenuiflorum* using common-garden morphology, whole chloroplast genomes, nuclear ITS, and ISSR markers. It also reports the first use of ISSR markers to assess genetic diversity in *Pediomelum*. This study demonstrates that *P. tenuiflorum* harbors substantial morphological and genetic variation, but it also demonstrates that this variation does not sort into well-supported monophyletic lineages corresponding to previously recognized morphotypes or geographic populations. Nuclear ITS data recovered *P. tenuiflorum* as a single clade with little internal resolution, while plastid phylogenies show deeply structured clades in which multiple *Pediomelum* species are nested, and ISSR markers detected polymorphism, but the exploratory ISSR dataset did not reveal obvious clustering in the banding patterns examined. The combined evidence points to extensive crossing among *P. tenuiflorum* morphotypes and hybridization with close *Pediomelum* relatives, leading to chloroplast capture, backcrossing, and cytonuclear discordance that complicate taxonomic delimitation.

Given these patterns, splitting *P. tenuiflorum* into multiple species based on the characters and markers analyzed here is not supported; instead, *P. tenuiflorum* is best interpreted as a single, morphologically variable species embedded in a reticulate evolutionary network with its congeners. More broadly, these results highlight the need for a denser phylogeny of this portion of *Pediomelum*, including multiple accessions per closely related species, to better resolve species boundaries and evaluate the extent of hybridization, introgression, and chloroplast capture across the genus. Future research leveraging cytogenetics and genome-scale nuclear markers will be essential for tracing specific hybridization pathways, testing for polyploidy, and refining species boundaries within *Pediomelum*, thereby providing a more complete understanding of how hybridization and gene flow shape diversity in this group.

## Data Availability

Sequence alignments were uploaded to Dryad (www.datadryad.org; alignments available from the Dryad Digital Repository [doi: 10.5061/dryad.n8pk0p397]). All new DNA sequences were uploaded to GenBank (accession numbers in Table S4).

## Supplementary Materials

The following supporting information can be downloaded at: https://www.mdpi.com/article/10.3390/genes17040490/s1, Table S1. Seventy-one *Pediomelum* samples included in the study, the herbarium from which it was borrowed or from the common garden (CG), and the location/county from which the sample was collected. * = samples used in ISSR study. Table S2. Averages of the morphological measurements made on 30 samples of *Pediomelum tenuiflorum* from the common garden and the locations where the seeds were collected. Table S3. Sequence read depths for chloroplast and nuclear markers for 71 samples of *Pediomelum* used in the phylogenies. Table S4. GenBank Accession Numbers for 71 samples of *Pediomelum* used in the phylogenies. Table S5. Eleven ISSRs primers used for the amplification of *Pediomelum tenuiflorum* including total amplified bands produced and total polymorphic bands. Figure S1. Phylogeny of *Pediomelum tenuiflorum* estimated from the chloroplast coding regions. Figure S2. Gel converted to linear plots for TGC_6_ Primer. Figure S3. Gel converted to linear plots for AG_8_ Primer. Figure S4. Gel converted to linear plots for AG_8_C Primer. Figure S5. Gel converted to linear plots for AGT_8_G Primer. Figure S6. Gel converted to linear plots for CTC_6_C Primer. Figure S7. Gel converted to linear plots for GTT_6_T Primer. Figure S8. Gel converted to linear plots for TC_8_A Primer. Figure S9. Gel converted to linear plots for TGC_6_G Primer.
