# Supplementary material for: Genetic Diversity and Phylogenetic Relationships Among Accessions of Pediomelum tenuiflorum (Pursh) A.N. Egan
Source: Genes (Basel). 2026 Apr 20;17(4):490. doi: 10.3390/genes17040490 (PMC13115678; doi:10.3390/genes17040490)
Supplement: Supplementary file 1 [file genes-17-00490-s001.zip › genes-4198477-supplementary.pdf]

Table S1. Seventy-one *Pediomelum* samples included in the study, the herbarium from which it was borrowed or from the common garden (CG), and the location/county from which the sample was collected. * = samples used in ISSR study.

Genus	Specific epithet	Sample ID	Herbaria	State	Location/County
Pediomelum	*cuspidatum*	CA094	ARIZ[a]	Texas	Coryell
Pediomelum	*cuspidatum*	CA117	OKLA[b]	Texas	Wise
Pediomelum	*cuspidatum*	CA135	TEX/LL[c]	Texas	McLennan
Pediomelum	*piedmontanum*	CA073	GH[d]	Georgia	Columbia
Pediomelum	*reverchonii*	CA133	TEX/LL[c]	Texas	Tarrant
Pediomelum	*tenuiflorum*	CA001	CG[e]	Nebraska	9-mile prairie
Pediomelum	*tenuiflorum*	CA002*	CG[e]	Nebraska	Table rock
Pediomelum	*tenuiflorum*	CA003*	CG[e]	Nebraska	Stanton
Pediomelum	*tenuiflorum*	CA004	CG[e]	Nebraska	9-mile prairie
Pediomelum	*tenuiflorum*	CA005	CG[e]	Nebraska	Julesburg
Pediomelum	*tenuiflorum*	CA006	CG[e]	Nebraska	Julesburg
Pediomelum	*tenuiflorum*	CA007	CG[e]	Nebraska	737/50SE
Pediomelum	*tenuiflorum*	CA008*	CG[e]	Nebraska	9-mile prairie
Pediomelum	*tenuiflorum*	CA009*	CG[e]	Nebraska	9-mile prairie
Pediomelum	*tenuiflorum*	CA010*	CG[e]	Nebraska	Julesburg
Pediomelum	*tenuiflorum*	CA013	CG[e]	Nebraska	737/50SE
Pediomelum	*tenuiflorum*	CA014	CG[e]	Nebraska	737/50SE
Pediomelum	*tenuiflorum*	CA015	CG[e]	Nebraska	Julesburg

Genus	Specific epithet	Sample ID	Herbaria	State	Location/County
Pediomelum	*tenuiflorum*	CA016	CG[e]	Nebraska	Stanton
Pediomelum	*tenuiflorum*	CA017	CG[e]	Nebraska	9-mile prairie
Pediomelum	*tenuiflorum*	CA018	CG[e]	Nebraska	Stanton
Pediomelum	*tenuiflorum*	CA019*	CG[e]	Nebraska	737/50SE
Pediomelum	*tenuiflorum*	CA020*	CG[e]	Nebraska	737/50SE
Pediomelum	*tenuiflorum*	CA021*	CG[e]	Nebraska	9-mile prairie
Pediomelum	*tenuiflorum*	CA022	CG[e]	Nebraska	Julesburg
Pediomelum	*tenuiflorum*	CA023	CG[e]	Nebraska	Table rock
Pediomelum	*tenuiflorum*	CA024	CG[e]	Nebraska	Stanton
Pediomelum	*tenuiflorum*	CA025	CG[e]	Nebraska	Stanton
Pediomelum	*tenuiflorum*	CA026	CG[e]	Nebraska	Table rock
Pediomelum	*tenuiflorum*	CA028	CG[e]	Nebraska	Stanton
Pediomelum	*tenuiflorum*	CA029	CG[e]	Nebraska	Jeff. Resv. Near
Pediomelum	*tenuiflorum*	CA031*	CG[e]	Nebraska	Julesburg
Pediomelum	*tenuiflorum*	CA033	CG[e]	Nebraska	N/A
Pediomelum	*tenuiflorum*	HR001	CG[e]	Nebraska	737/50SE
Pediomelum	*tenuiflorum*	HR002	CG[e]	Nebraska	Jeff. Resv. Near
Pediomelum	*tenuiflorum*	HR003	CG[e]	Nebraska	Stanton
Pediomelum	*tenuiflorum*	HR004	CG[e]	Nebraska	Jeff. Resv. Upper
Pediomelum	*tenuiflorum*	CA035*	MIN[f]	Iowa	Union

Genus	Specific epithet	Sample ID	Herbaria	State	Location/County
Pediomelum	*tenuiflorum*	CA036	KSC[g]	Kansas	Morton
Pediomelum	*tenuiflorum*	CA045	ARIZ[a]	Arizona	Santa Cruz
Pediomelum	*tenuiflorum*	CA047	TEX/LL[c]	Texas	Lamar
Pediomelum	*tenuiflorum*	CA050*	RM[h]	Wyoming	Sheridan
Pediomelum	*tenuiflorum*	CA054	KSC[g]	Kansas	Washington
Pediomelum	*tenuiflorum*	CA056*	KANU[i]	Colorado	El Paso
Pediomelum	*tenuiflorum*	CA063	MONTU[j]	Montana	Carbon
Pediomelum	*tenuiflorum*	CA064*	MONTU[j]	Montana	Fergus
Pediomelum	*tenuiflorum*	CA069	ILLS[k]	Illinois	Cass
Pediomelum	*tenuiflorum*	CA071	RM[h]	Wyoming	Campbell
Pediomelum	*tenuiflorum*	CA072	RM[h]	Wyoming	Converse
Pediomelum	*tenuiflorum*	CA076	SRP[l]	Nebraska	Dawes
Pediomelum	*tenuiflorum*	CA079	KSC[g]	Kansas	Saline
Pediomelum	*tenuiflorum*	CA080	KSC[g]	Kansas	Graham
Pediomelum	*tenuiflorum*	CA082	MIN[f]	Minnesota	Houston
Pediomelum	*tenuiflorum*	CA083	MIN[f]	Minnesota	Filmore
Pediomelum	*tenuiflorum*	CA086	KANU[i]	Kansas	Thomas
Pediomelum	*tenuiflorum*	CA088	KANU[i]	Kansas	Pratt
Pediomelum	*tenuiflorum*	CA089	ARIZ[a]	Arizona	Santa Cruz
Pediomelum	*tenuiflorum*	CA090	ARIZ[a]	Arizona	Yavapai

Genus	Specific epithet	Sample ID	Herbaria	State	Location/County
Pediomelum	*tenuiflorum*	CA091	ARIZ[a]	Arizona	Pinal
Pediomelum	*tenuiflorum*	CA092	ARIZ[a]	Arizona	Pinal
Pediomelum	*tenuiflorum*	CA107	RM[h]	Nebraska	Sioux
Pediomelum	*tenuiflorum*	CA109	RM[h]	Wyoming	Platte
Pediomelum	*tenuiflorum*	CA111	RM[h]	Wyoming	Niobrara
Pediomelum	*tenuiflorum*	CA112	RM[h]	Wyoming	Johnson
Pediomelum	*tenuiflorum*	CA113	OKLA[b]	Oklahoma	Osage
Pediomelum	*tenuiflorum*	CA114	OKLA[b]	Oklahoma	Beaver
Pediomelum	*tenuiflorum*	CA120	OKL[m]	Oklahoma	Harper
Pediomelum	*tenuiflorum*	CA121	OKL[m]	Oklahoma	Cimarron
Pediomelum	*tenuiflorum*	CA130	TEX/LL[c]	Texas	Palo Pinto
Pediomelum	*tenuiflorum*	CA134*	TEX/LL[c]	Texas	Palo Pinto
Pediomelum	*tenuiflorum*	CA139	TEX/LL[c]	Texas	Lamar

[a] University of Arizona
[b] Oklahoma State University
[c] University of Texas at Austin
[d] Harvard University
[e] Common Garden Nebraska
[f] University of Minnesota
[g] Kansas State University
[h] University of Wyoming
[i] University of Kansas
[j] University of Montana
[k] Illinois National History Survey
[l] Boise State University
[m] University of Oklahoma

* indicates samples used in ISSR study, four samples: CA0521, CA067, CA093, and CA119, were used in the ISSR study but not the phylogeny tree

Table S2. Averages of the morphological measurements made on 30 samples of *Pediomelum tenuiflorum* from the common garden and the locations where the seeds were collected.

Location	Stem Diameter(mm)	Stem Length(mm)	Corolla Length(mm)	Flower Internode Length(mm)	Leaflet Length (mm)	Leaflet Width (mm)
Julesburg	2.455	34	3.82	7.15	13.95	2.55
737/50SE	3.724	37	4.55	5.34	16.71	2.72
Stanton	2.723	37	N/A	5.34	14.93	2.87
Stanton	4.502	46	N/A	4.66	12.69	3.81
9-mile prairie	5.078	65	4.83	6.22	21.1	4.37
Stanton	3.331	49	N/A	4.86	15.53	3.26
737/50SE	4.03	49	4.45	4.87	15	3.61
Stanton	3.146	56	4.7	4.95	13.02	2.91
Jeff. Resv. Near	NA	57	4.86	10.75	13.44	2.78
Jeff. Resv. Near	NA	58	4.39	11.27	14.95	2.28
737/50SE	2.836	51	4.44	8.4	10.96	2.36
Julesburg	1.841	57	4	5.59	10.81	2.61
737/50SE	4.605	88	5.1	4.19	12.98	3.04

Location	Stem Diameter(mm)	Stem Length(mm)	Corolla Length(mm)	Flower Internode Length(mm)	Leaflet Length (mm)	Leaflet Width (mm)
737/50SE	4.292	62	N/A	5.8	17.53	3.46
Julesburg	2.848	38	4.82	8.85	12.27	2.78
9-mile prairie	2.586	38	4.96	5.86	15.42	3.01
Stanton	3.53	67	4.94	4.73	15.52	3.28
Table rock	5.26	57	N/A	6.83	16.62	2.92
Jeff. Resv. Near	4.097	87	4.13	10.64	14.28	2.86
737/50SE	5.562	90	5.06	4.44	12.62	3.52
Julesburg	3.924	52	4.63	8.27	11.02	2.7
9-mile prairie	3.615	48	4.81	5.12	16.6	3.53
737/50SE	2.815	40	N/A	4.69	17.54	3.62
Table rock	3.902	60	N/A	6.39	15.27	3.02
Stanton	2.946	46	4.92	4.93	14.36	3.29
Stanton	2.369	29	4.83	4.47	14.14	2.65
Julesburg	2.017	28	4.94	8.97	11.16	3.7
Jeff. Resv. Near	NA	40	4.02	8.4	9.45	2.49

Location	Stem Diameter(mm)	Stem Length(mm)	Corolla Length(mm)	Flower Internode Length(mm)	Leaflet Length (mm)	Leaflet Width (mm)
Stanton	NA	33	N/A	4.39	11.9	2.79
737/50SE	4.507	60	N/A	6.18	17.37	3.7
Jeff. Resv. Upper	NA	36	4.18	4.24	13.66	3.48

Table S3. Sequence read depths for chloroplast and nuclear markers for 71 samples of *Pediomelum* used in the phylogenies.

Genus	Specific epithet	Sample ID	Depth reads cpDNA	Depth reads ITS
Pediomelum	*cuspidatum*	CA094	2229	1485
Pediomelum	*cuspidatum*	CA117	509	2007
Pediomelum	*cuspidatum*	CA135	1117	2200
Pediomelum	*piedmontanum*	CA073	380	3321
Pediomelum	*reverchonii*	CA133	11364	10724
Pediomelum	*tenuiflorum*	CA001	824	4029
Pediomelum	*tenuiflorum*	CA002	218	1599
Pediomelum	*tenuiflorum*	CA003	602	2554
Pediomelum	*tenuiflorum*	CA004	597	2411
Pediomelum	*tenuiflorum*	CA005	319	1965
Pediomelum	*tenuiflorum*	CA006	622	5291
Pediomelum	*tenuiflorum*	CA007	509	6837
Pediomelum	*tenuiflorum*	CA008	4026	21454
Pediomelum	*tenuiflorum*	CA009	4168	39877
Pediomelum	*tenuiflorum*	CA010	937	3316
Pediomelum	*tenuiflorum*	CA013	415	1406
Pediomelum	*tenuiflorum*	CA014	874	5850
Pediomelum	*tenuiflorum*	CA015	768	4935
Pediomelum	*tenuiflorum*	CA016	402	3686
Pediomelum	*tenuiflorum*	CA017	911	2488
Pediomelum	*tenuiflorum*	CA018	528	2062
Pediomelum	*tenuiflorum*	CA019	566	6846
Pediomelum	*tenuiflorum*	CA020	450	2506
Pediomelum	*tenuiflorum*	CA021	709	2993
Pediomelum	*tenuiflorum*	CA022	700	2799
Pediomelum	*tenuiflorum*	CA023	646	2412

**Genus**	**Specific epithet**	**Sample ID**	**Depth reads cpDNA**	**Depth reads ITS**
Pediomelum	*tenuiflorum*	CA024	670	3466
Pediomelum	*tenuiflorum*	CA025	692	4086
Pediomelum	*tenuiflorum*	CA026	4408	15300
Pediomelum	*tenuiflorum*	CA028	294	2728
Pediomelum	*tenuiflorum*	CA029	599	3083
Pediomelum	*tenuiflorum*	CA031	872	5287
Pediomelum	*tenuiflorum*	CA033	965	1834
Pediomelum	*tenuiflorum*	CA035	5185	3917
Pediomelum	*tenuiflorum*	CA036	2879	1168
Pediomelum	*tenuiflorum*	CA045	6946	5625
Pediomelum	*tenuiflorum*	CA047	726	946
Pediomelum	*tenuiflorum*	CA050	1430	4677
Pediomelum	*tenuiflorum*	CA054	2538	1616
Pediomelum	*tenuiflorum*	CA056	3694	2144
Pediomelum	*tenuiflorum*	CA063	1507	761
Pediomelum	*tenuiflorum*	CA064	2094	2806
Pediomelum	*tenuiflorum*	CA069	3565	1525
Pediomelum	*tenuiflorum*	CA071	1652	1847
Pediomelum	*tenuiflorum*	CA072	2234	2515
Pediomelum	*tenuiflorum*	CA076	17822	8202
Pediomelum	*tenuiflorum*	CA079	2515	1691
Pediomelum	*tenuiflorum*	CA080	3593	1796
Pediomelum	*tenuiflorum*	CA081	2069	3378
Pediomelum	*tenuiflorum*	CA082	3653	2586
Pediomelum	*tenuiflorum*	CA083	645	2573
Pediomelum	*tenuiflorum*	CA086	4858	917
Pediomelum	*tenuiflorum*	CA088	3247	1341
Pediomelum	*tenuiflorum*	CA089	6369	2785

Genus	Specific epithet	Sample ID	Depth reads cpDNA	Depth reads ITS
Pediomelum	*tenuiflorum*	CA090	3745	2219
Pediomelum	*tenuiflorum*	CA091	1166	399
Pediomelum	*tenuiflorum*	CA093	4545	16332
Pediomelum	*tenuiflorum*	CA107	1634	1872
Pediomelum	*tenuiflorum*	CA109	10131	7419
Pediomelum	*tenuiflorum*	CA111	1044	2091
Pediomelum	*tenuiflorum*	CA112	1270	1525
Pediomelum	*tenuiflorum*	CA113	798	1224
Pediomelum	*tenuiflorum*	CA114	1029	951
Pediomelum	*tenuiflorum*	CA120	7938	3880
Pediomelum	*tenuiflorum*	CA121	4296	2526
Pediomelum	*tenuiflorum*	CA130	837	561
Pediomelum	*tenuiflorum*	CA134	1872	1614
Pediomelum	*tenuiflorum*	CA139	3385	801
Pediomelum	*tenuiflorum*	HR001	475	2648
Pediomelum	*tenuiflorum*	HR002	976	7728
Pediomelum	*tenuiflorum*	HR003	650	6200
Pediomelum	*tenuiflorum*	HR004	538	4572
Pediomelum	*tenuiflorum*	JN1026	1582	3010
Pediomelum	*tenuiflorum*	SU8144	1647	1629
Pediomelum	*tenuiflorum*	SU8317	785	2083
Pediomelum	*argophyllum*	JN1027	1779	3426
Pediomelum	*digitatum*	JN1022	665	1757
Pediomelum	*esculentum*	AH014	962	7537

Table S4: GenBank Accession Numbers for 71 samples of *Pediomelum* used in the phylogenies.

Genus	Specific epithet	Sample ID	ITS Accession No	Chloroplast Accession No
Pediomelum	*cuspidatum*	CA094	PZ224753	PZ268291
Pediomelum	*cuspidatum*	CA117	PZ224755	PZ268292
Pediomelum	*cuspidatum*	CA135	PZ224754	PZ268293
Pediomelum	*piedmontanum*	CA073	PZ224756	PZ268361
Pediomelum	*reverchonii*	CA133	PZ224757	PZ268294
Pediomelum	*tenuiflorum*	CA001	PZ224759	PZ268296
Pediomelum	*tenuiflorum*	CA002	PZ224760	PZ268297
Pediomelum	*tenuiflorum*	CA003	PZ224761	PZ268298
Pediomelum	*tenuiflorum*	CA004	PZ224762	PZ268299
Pediomelum	*tenuiflorum*	CA005	PZ224763	PZ268300
Pediomelum	*tenuiflorum*	CA006	PZ224764	PZ268301
Pediomelum	*tenuiflorum*	CA007	PZ224765	PZ268302
Pediomelum	*tenuiflorum*	CA008	PZ224766	PZ268303
Pediomelum	*tenuiflorum*	CA009	PZ224767	PZ268304
Pediomelum	*tenuiflorum*	CA010	PZ224768	PZ268305
Pediomelum	*tenuiflorum*	CA013	PZ224769	PZ268306
Pediomelum	*tenuiflorum*	CA014	PZ224770	PZ268307
Pediomelum	*tenuiflorum*	CA015	PZ224771	PZ268308
Pediomelum	*tenuiflorum*	CA016	PZ224772	PZ268309

**Genus**	**Specific epithet**	**Sample ID**	**ITS Accession No**	**Chloroplast Accession No**
Pediomelum	*tenuiflorum*	CA017	PZ224773	PZ268310
Pediomelum	*tenuiflorum*	CA018	PZ224774	PZ268311
Pediomelum	*tenuiflorum*	CA019	PZ224775	PZ268312
Pediomelum	*tenuiflorum*	CA020	PZ224776	PZ268313
Pediomelum	*tenuiflorum*	CA021	PZ224777	PZ268314
Pediomelum	*tenuiflorum*	CA022	PZ224778	PZ268315
Pediomelum	*tenuiflorum*	CA023	PZ224779	PZ268316
Pediomelum	*tenuiflorum*	CA024	PZ224780	PZ268317
Pediomelum	*tenuiflorum*	CA025	PZ224781	PZ268318
Pediomelum	*tenuiflorum*	CA026	PZ224782	PZ268319
Pediomelum	*tenuiflorum*	CA028	PZ224783	PZ268320
Pediomelum	*tenuiflorum*	CA029	PZ224784	PZ268321
Pediomelum	*tenuiflorum*	CA031	PZ224785	PZ268322
Pediomelum	*tenuiflorum*	CA033	PZ224758	PZ268295
Pediomelum	*tenuiflorum*	CA036	PZ224786	PZ268323
Pediomelum	*tenuiflorum*	CA045	PZ224787	PZ268324
Pediomelum	*tenuiflorum*	CA047	PZ224788	PZ268325
Pediomelum	*tenuiflorum*	CA050	PZ224789	PZ268326
Pediomelum	*tenuiflorum*	CA054	PZ224790	PZ268327
Pediomelum	*tenuiflorum*	CA056	PZ224791	PZ268328

**Genus**	**Specific epithet**	**Sample ID**	**ITS Accession No**	**Chloroplast Accession No**
Pediomelum	*tenuiflorum*	CA063	PZ224792	PZ268329
Pediomelum	*tenuiflorum*	CA064	PZ224793	PZ268330
Pediomelum	*tenuiflorum*	CA069	PZ224794	PZ268331
Pediomelum	*tenuiflorum*	CA071	PZ224795	PZ268332
Pediomelum	*tenuiflorum*	CA072	PZ224796	PZ268333
Pediomelum	*tenuiflorum*	CA076	PZ224797	PZ268334
Pediomelum	*tenuiflorum*	CA079	PZ224798	PZ268335
Pediomelum	*tenuiflorum*	CA080	PZ224799	PZ268336
Pediomelum	*tenuiflorum*	CA081	PZ224800	PZ268337
Pediomelum	*tenuiflorum*	CA082	PZ224801	PZ268338
Pediomelum	*tenuiflorum*	CA083	PZ224802	PZ268339
Pediomelum	*tenuiflorum*	CA086	PZ224803	PZ268340
Pediomelum	*tenuiflorum*	CA088	PZ224804	PZ268341
Pediomelum	*tenuiflorum*	CA089	PZ224805	PZ268342
Pediomelum	*tenuiflorum*	CA090	PZ224806	PZ268343
Pediomelum	*tenuiflorum*	CA091	PZ224807	PZ268344
Pediomelum	*tenuiflorum*	CA093	PZ224808	PZ268345
Pediomelum	*tenuiflorum*	CA107	PZ224809	PZ268346
Pediomelum	*tenuiflorum*	CA109	PZ224810	PZ268347
Pediomelum	*tenuiflorum*	CA111	PZ224811	PZ268348

**Genus**	**Specific epithet**	**Sample ID**	**ITS Accession No**	**Chloroplast Accession No**
Pediomelum	*tenuiflorum*	CA112	PZ224812	PZ268349
Pediomelum	*tenuiflorum*	CA113	PZ224813	PZ268350
Pediomelum	*tenuiflorum*	CA114	PZ224814	PZ268351
Pediomelum	*tenuiflorum*	CA120	PZ224815	PZ268352
Pediomelum	*tenuiflorum*	CA121	PZ224816	PZ268353
Pediomelum	*tenuiflorum*	CA130	PZ224817	PZ268354
Pediomelum	*tenuiflorum*	CA134	PZ224818	PZ268355
Pediomelum	*tenuiflorum*	CA139	PZ224819	PZ268356
Pediomelum	*tenuiflorum*	HR001	PZ224820	PZ268357
Pediomelum	*tenuiflorum*	HR002	PZ224821	PZ268358
Pediomelum	*tenuiflorum*	HR003	PZ224822	PZ268359
Pediomelum	*tenuiflorum*	HR004	PZ224823	PZ268360

Table S5. Eleven ISSRs primers used for the amplification of *Pediomelum tenuiflorum* including total amplified bands produced and total polymorphic bands.

Primers	No of amplified bands	No. of Polymorphic bands	% polymorphism
AC_8G	21	8	38.10
AG_8	55	10	18.18
AG_8C	56	7	12.5
AGT_6G	18	3	16.67
CTC_6A	63	11	17.46
CTC_6C	23	4	17.39
GTT_6T	29	3	10.34
TC_8A	8	2	25
TGC_6	0	0	0
TGC_6C	37	3	8.11
TGC_6G	35	10	28.57
**Total**	**345**	**61**	**17.68**

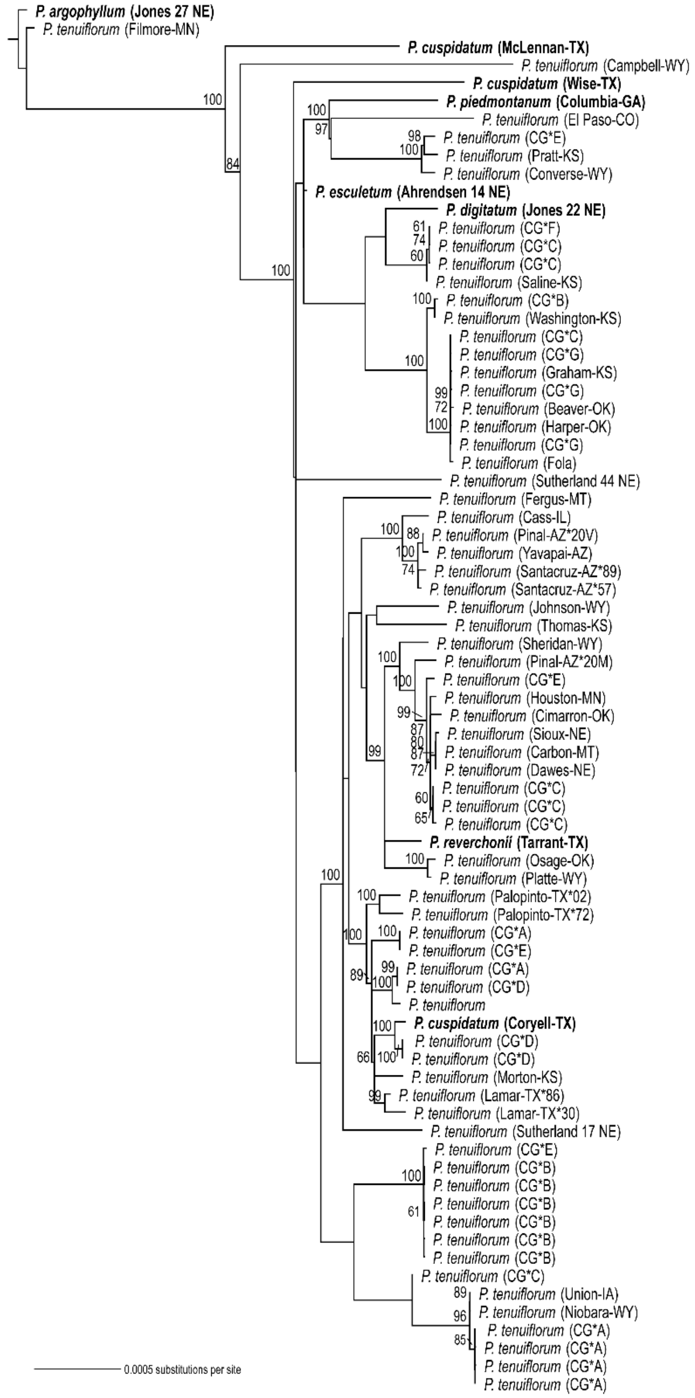

Figure S1. Phylogeny of *Pediomelum tenuiflorum* estimated from the chloroplast coding regions. Maximum likelihood (ML) phylogeny (-ln L = 124089.89), which includes 77 samples with *P. argophyllum* as the outgroup. Species other than *P. tenuiflorum* are in bold text. Collection locations (county-state) are shown in parentheses for each sample. Samples from the common garden are indicated with CG* and the Nebraska (except one) location from which their seeds were collected, as follows: A = Nine-mile prairie, B = Stanton County, C = Intersection of Hwy 737&50, D = Table Rock Wildlife Management Area, E = Julesburg, Colorado, F = Jeffrey Reservoir (upper), and G = Jeffrey Reservoir (near). Samples from the same location and county are indicated by * and the last two digits of the collector's number, and if they have the same last two digits, the first letter of the collector's last name is included. Numbers above branches indicate ML bootstrap (BS) values resulting from 1000 replicates. If no BS values are shown, BS ≤ 50.

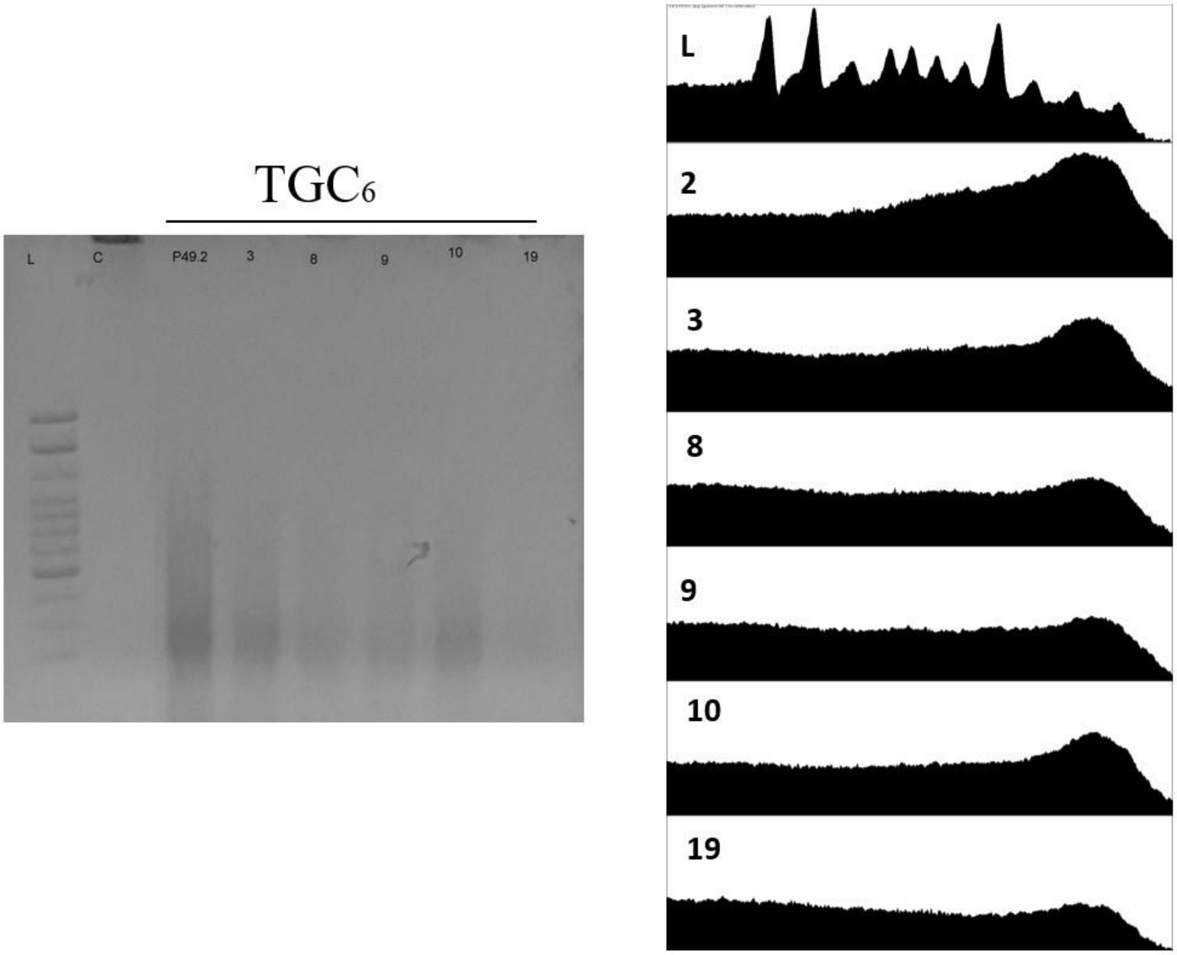

Figure S2. Gel converted to linear plots for TGC_6 Primer. Lanes represent each sample of *P. tenuiflorum* used. The number on linear plot corresponds with the lanes, with all samples here representing the morphotypes from the common garden. The TGC_6 primer had no amplification of any *P. tenuiflorum* as clusters formed on linear plots were smudges from the running of the gel.

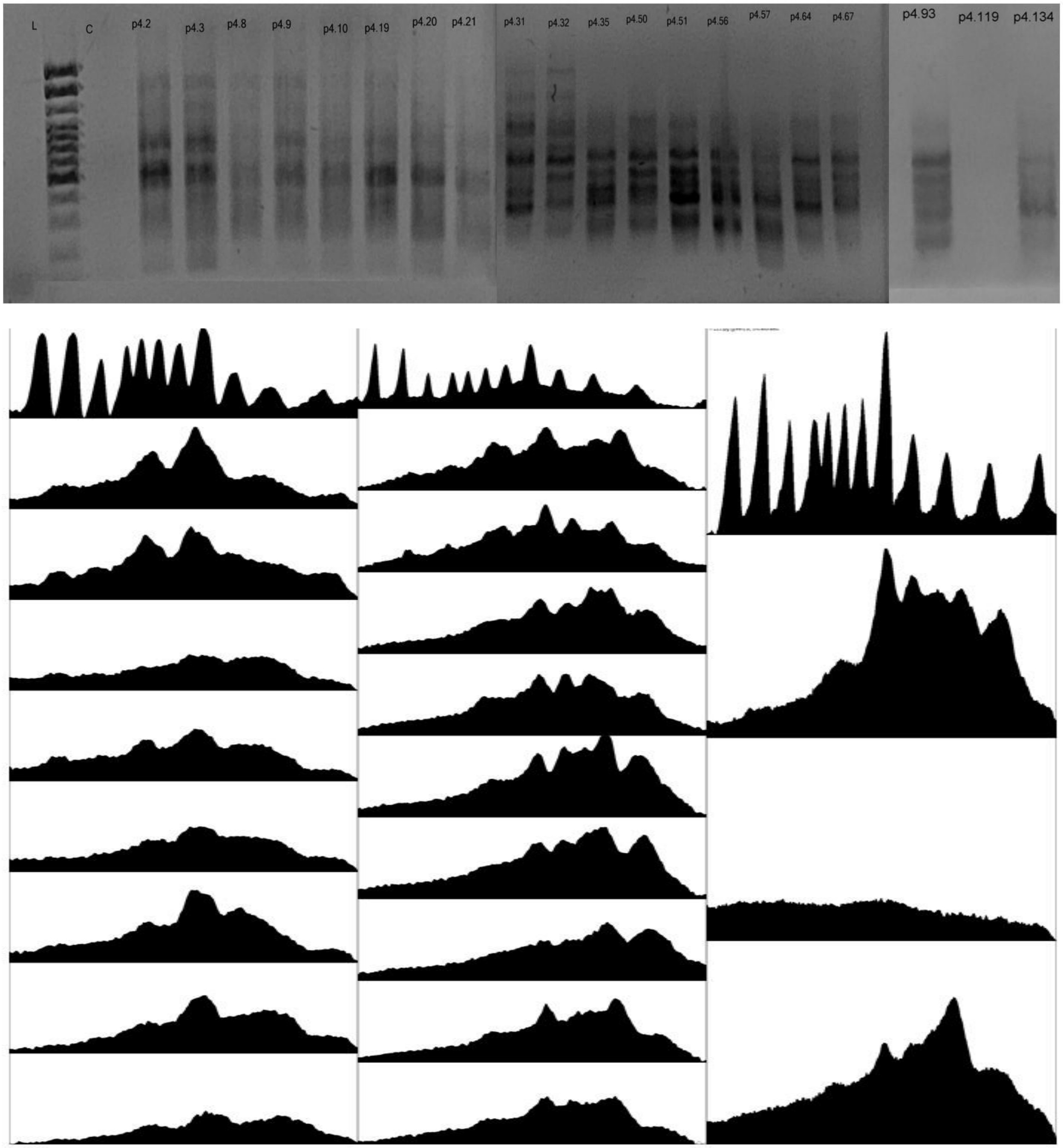

Figure S3. Gel converted to linear plots for AG_8 Primer. Lanes represent each sample of *P. tenuiflorum* used. The number on the linear plot corresponds with the lanes, with all samples here representing the morphotypes from the common garden. The AG_8 primer had amplification of almost all the *P. tenuiflorum*, and some clusters formed on linear plots were smudges from the running of the gel.

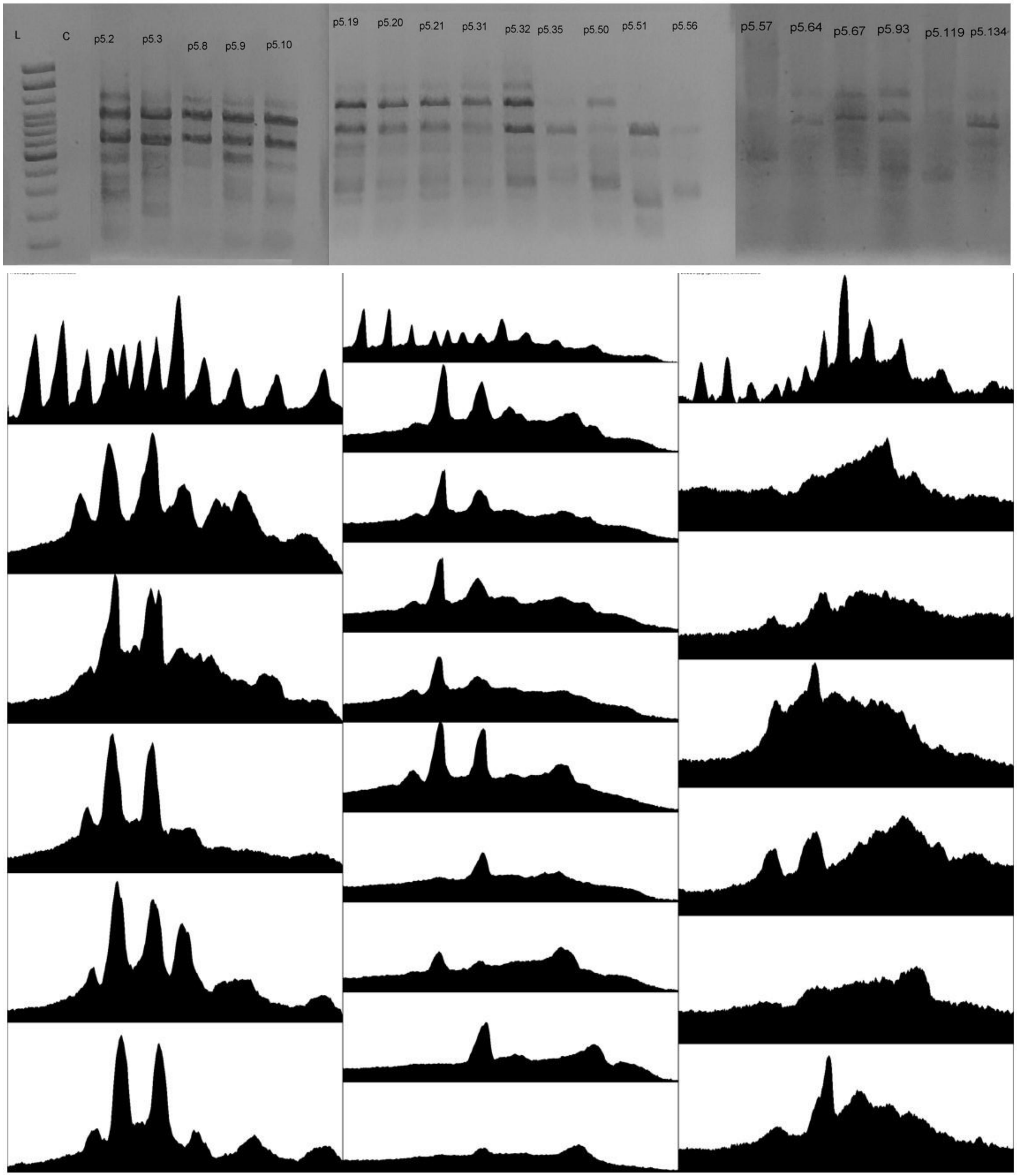

Figure S4. Gel converted to linear plots for AG$_8$C Primer. Lanes represent each sample of *P. tenuiflorum* used. The number on the linear plot corresponds with the lanes, with all samples here representing the morphotypes from the common garden. The AG$_8$C primer had the second-highest amplification of all *P. tenuiflorum*.

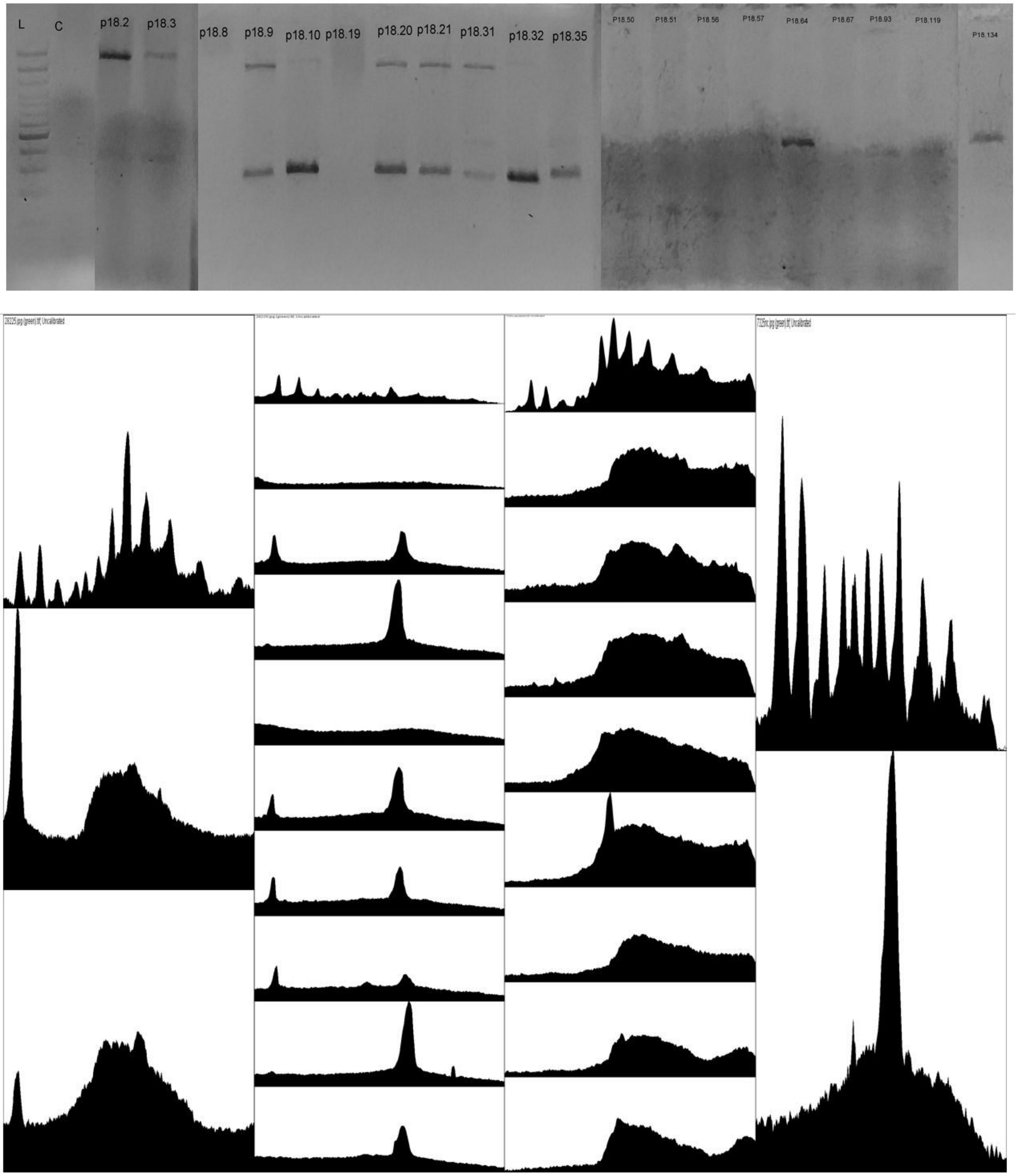

Figure S5. Gel converted to linear plots for AGT$_8$G Primer. Lanes represent each sample of *P. tenuiflorum* used. The number on the linear plot corresponds with the lanes, with all samples here representing the morphotypes from the common garden. The AGT$_8$G primer had very few amplifications of the *P. tenuiflorum,* and some clusters formed on linear plots were smudges from the running of the gel.

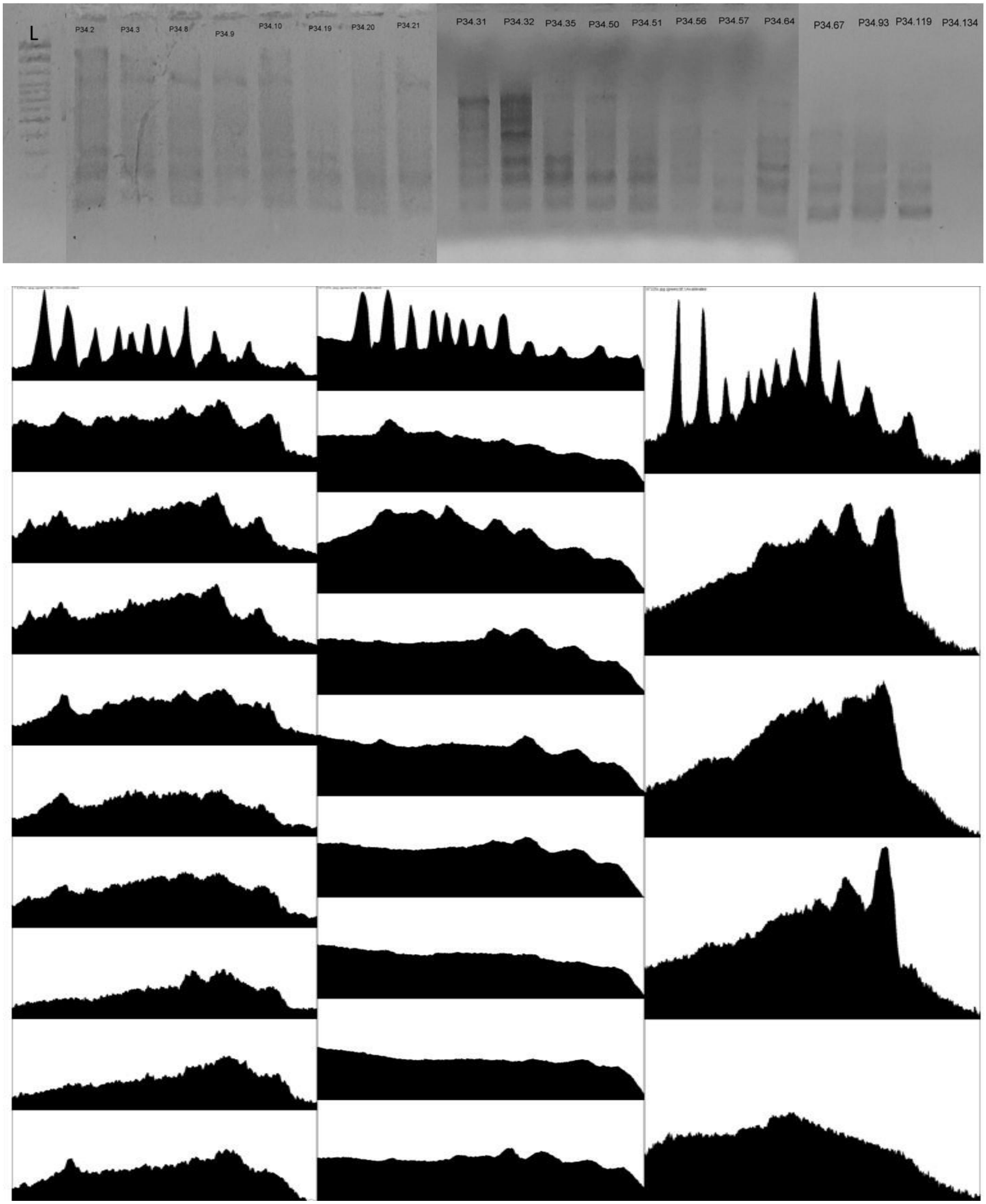

Figure S6. Gel converted to linear plots for CTC$_6$C Primer. Lanes represent each sample of *P. tenuiflorum* used. The number on the linear plot corresponds with the lanes, with all samples here representing the morphotypes from the common garden. The CTC$_6$C primer had amplifications of *P. tenuiflorum*, and some clusters formed on linear plots were smudges from the running of the gel.

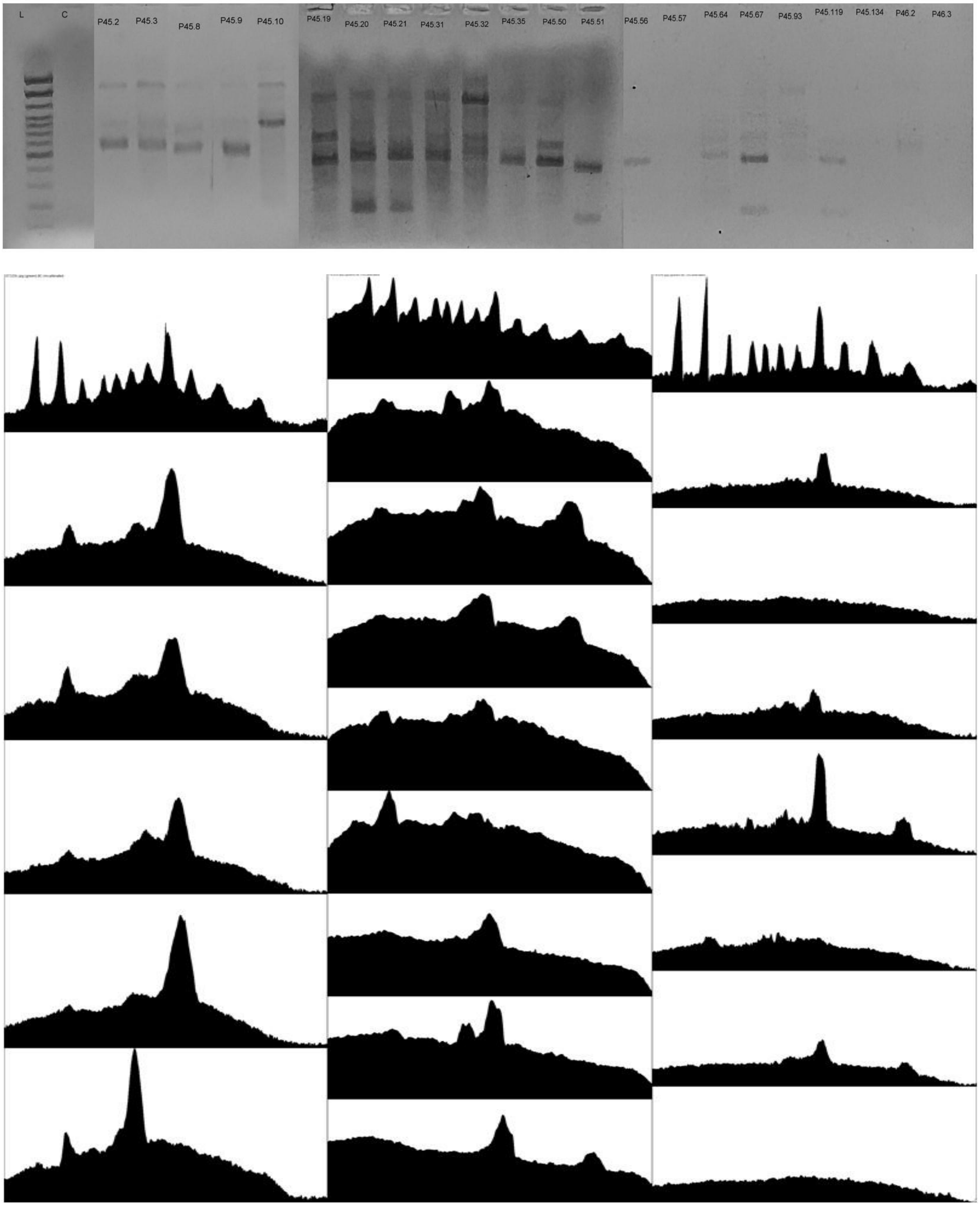

Figure S7. Gel converted to linear plots for GTT$_6$T Primer. Lanes represent each sample of *P. tenuiflorum* used. The number on the linear plot corresponds with the lanes, with all samples here representing the morphotypes from the common garden. The GTT$_6$T primer had good amplification of *P. tenuiflorum*.

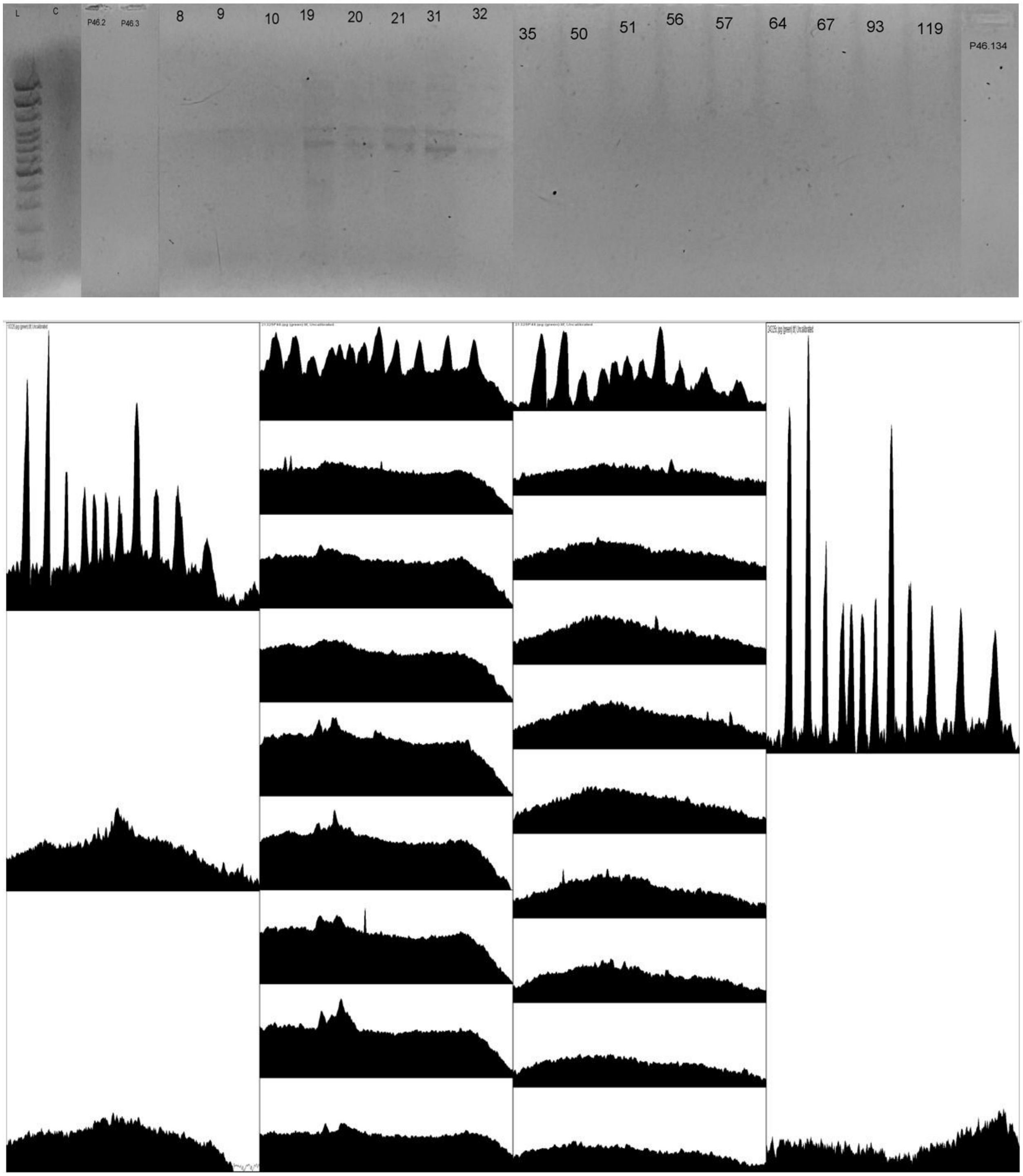

Figure S8. Gel converted to linear plots for TC_8A Primer. Lanes represent each sample of *P. tenuiflorum* used. The number on the linear plot corresponds with the lanes, with all samples here representing the morphotypes from the common garden. The TC_8A primer had very few amplifications of *P. tenuiflorum*.

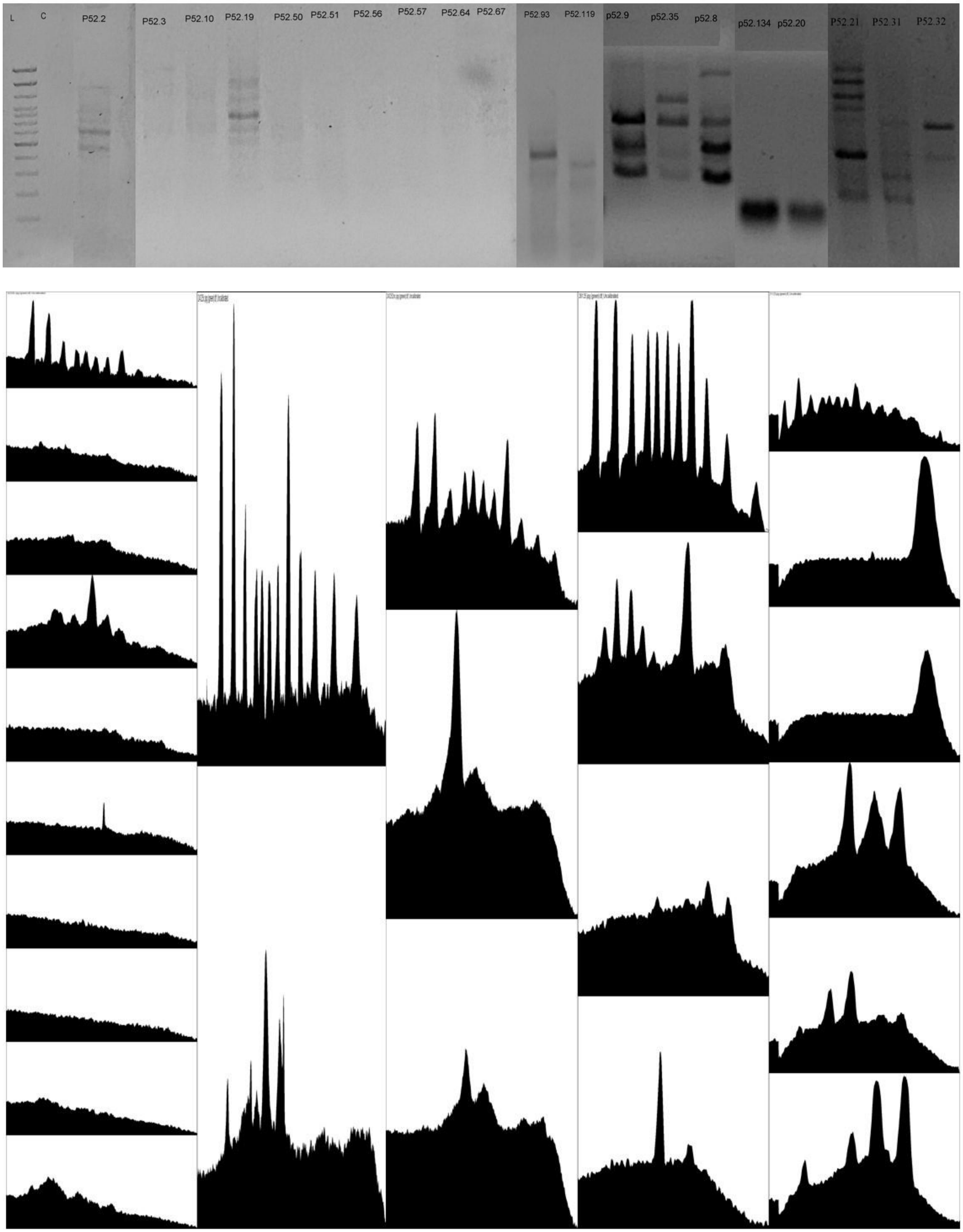

Figure S9. Gel converted to linear plots for TGC$_6$G Primer. Lanes represent each sample of *P. tenuiflorum* used. The number on the linear plot corresponds with the lanes, with all samples here representing the morphotypes from the common garden. The TGC$_6$G primer had good amplifications of *P. tenuiflorum*.
